# Artificial spinning of natural silk threads

**DOI:** 10.1038/s41598-019-51589-9

**Published:** 2019-10-28

**Authors:** Martin Frydrych, Alexander Greenhalgh, Fritz Vollrath

**Affiliations:** Department of Zoology, University of Oxford, Mansfield Road, Oxford, OX1 3SZ United Kingdom

**Keywords:** Biomaterials, Biomaterials - proteins, Biopolymers, Mechanical properties, Biomaterials

## Abstract

Silk producing arthropods spin solid fibres from an aqueous protein feedstock apparently relying on the complex structure of the silk protein and its controlled aggregation by shear forces, alongside biochemical changes. This flow-induced phase-transition of the stored native silk molecules is irreversible, environmentally sound and remarkably energy efficient. The process seemingly relies on a self-assembling, fibrillation process. Here we test this hypothesis by biomimetically spinning a native-based silk feedstock, extracted by custom processes, into silk fibres that equal their natural models’ mechanical properties. Importantly, these filaments, which featured cross-section morphologies ranged from large crescent-like to small ribbon-like shapes, also had the slender cross-sectional areas of native fibres and their hierarchical nanofibrillar structures. The modulation of the post-draw conditions directly affected mechanical properties, correlated with the extent of fibre crystallinity, i.e. degree of molecular order. We believe our study contributes significantly to the understanding and development of artificial silks by demonstrating successful biomimetic spinning relies on appropriately designed feedstock properties. In addition, our study provides inspiration for low-energy routes to novel synthetic polymers.

## Introduction

Natural silk fibres are spun in a highly efficient stress-induced and pH-facilitated pultrusion process that first orientates and then denatures the constituent fibroin protein molecules into self-assembled nanofibrils that combine to form the fibre thread^1^. Pultrusion speed and post-draw stretching affects the degree of molecular order and thus allows the animal to constantly tune its thread to its requirements by balancing the mechanical traits of stress and strain^2,3^. It is this combination of achieving substantial strength and considerable elongation simply by modulating the draw conditions of a single feedstock that makes natural silks not only interesting materials but also valuable models for the design of next-generation synthetic polymers^4,5^. The animal, be it spider or silkworm, stores the spinning dope as a viscous gel, we term an aquamelt, that solidifies rapidly by expelling its constituent water molecules during flow-shear in the tapering duct^4,5^. Importantly, the associated phase transition is not only orders of magnitude more energetically efficient than processing standard synthetic polymers, but it is also irreversible once completed^4^. In this context, prior attempts at creating and spinning artificial silks did not manage to utilize the low energy, irreversible flow induced phase transition of silk via true pultrusion, a key capability so far lacking in gene-engineered silks^6^. In order to study the aquamelt spinning process *ex-vivo*, one requires sufficient dope material for conducting appropriate spinning test-runs. This is not without challenges as the dope is exceptionally shear sensitive^4,5^. Here, we present an efficient and effective extraction method to produce bulk quantities of functional native-based silk dope. We further demonstrate that this dope can be spun into respectable fibres in a simple bioinspired water-based process. In Nature, silk is spun by pultrusion from dopes that are highly concentrated e.g. ~24% w/w^1^. Here we show that the extracted dope, which is as close to native as possible, can be artificially spun at protein concentrations as low as 6.4% w/w. This unexpected discovery allowed us to explore the silk’s natural and energy efficient aquamelt fibrillation process with its intrinsic stress-induction and pH-annealing.

## Results and Discussion

**Figure 1:**
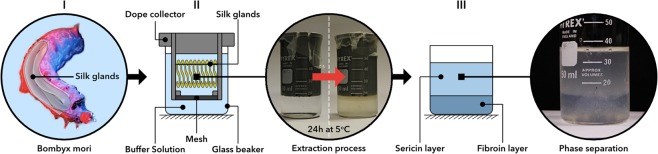
Extraction steps to obtain NSB Solution. (I) Briefly, native silk glands from *Bombyx mori* silkworms (5^th^ instar, after final excretion) were dissected, washed and the anterior and posterior middle gland trimmed. (II) The prepared glands were hung on wires (at the hairpin loop of the posterior middle sections; 18 glands were used for each extraction process) in a custom-made dope collector, which was placed within extraction buffer filled beaker. The loaded dope collector was then stored at 5 °C overnight, allowing a gravity assisted extraction. (III) The dope collector was removed, resulting in a two-phase solution, which could be separated and filtered.

**Figure 2:**
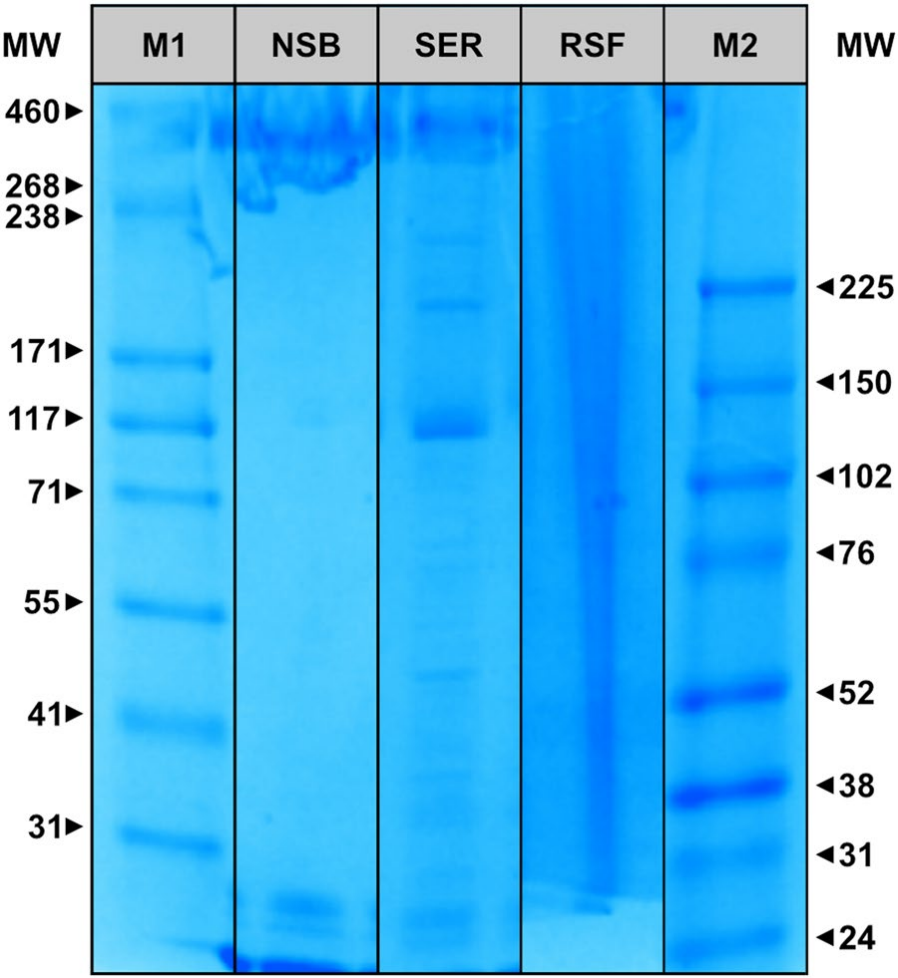
SDS-PAGE analysis of NSB, SER and RSF solution (MW: Molecular weight; M1: HiMark protein standard (31–460 kDa); NSB: Extracted fibroin layer; SER: Extracted sericin layer; RSF: Regenerated silk fibroin; M2: Rainbow protein standard (24–225 kDa)). *Note*: Samples are cropped from two gels, full-length gels are presented in Supplementary Fig. S1.

Standard liquid silk regeneration and extraction techniques either result in low quality, denatured and lower molecular weight dopes, or produces quantities only suitable for basic analyses, respectively^5–9^. By suspending minimally processed *Bombyx mori* glands into an aqueous, pH-controlled ammonium acetate solution (10 mM; pH 7.3 at 10 °C; adjusted with a 1% v/v ammonia solution), gland contents can be directly extracted under gravity into a two-phase solution of silk fibroin and sericin, without a regeneration step (Fig. 1)^6^. The upper phase-separated sericin layer (SER) can be removed, leaving a lower phase of pure silk fibroin. Handling is improved, and stability maintained, without protein aggregation, for at least one week in this native-based silk buffer (NSB). Long-term storage of the transparent NSB solution resulted in aggregation (gelation), characterised by an opaque appearance and rigid gel structure. SDS-PAGE electrophoresis showed no molecular degradation of NSB (Fig. 2, Supplementary Information: Fig. S1), with distinct bands directly comparable to traditionally dissected silk, lacking the distinctive smearing of regenerated silk fibroins (RSFs)^7^. Briefly, NSB samples were characterised with a high molecular weight band between 268 to 460 kDa, associated to heavy chains (~370 kDa), and featured two low molecular weight bands between 25 to 31 kDa, which can be associated with light chains (~25 kDa) and P25 (~27 kDa). By comparison, RSF samples presented extensive smearing and featured no identifiable bands in the low and high molecular weight regions, demonstrating a high degree of protein degradation, as expected, due to the harsh degumming and regeneration processes^8,10^. In respect to SER samples, SDS-PAGE electrophoresis presented several distinctive bands within a wide range of molecular weight of 31 to 268 kDa, which are generally not detectable in degummed sericin^11,12^. Importantly, these bands are not identifiable in the NSB samples, suggesting relatively high purity, which was also confirmed through FTIR (Supplementary Information: Fig. S2). However, the sericin samples showed visible bands in the low and high molecular weight regions of 24 to 31 kDa and 268 to 460 kDa, respectively, resembling bands present in NSB and could reflect unilateral contamination. Nonetheless, the extracted sericin enables the analysis of natively conformed sericin proteins, undamaged by degumming regimes, which will be further pursued in the future.

**Figure 3:**
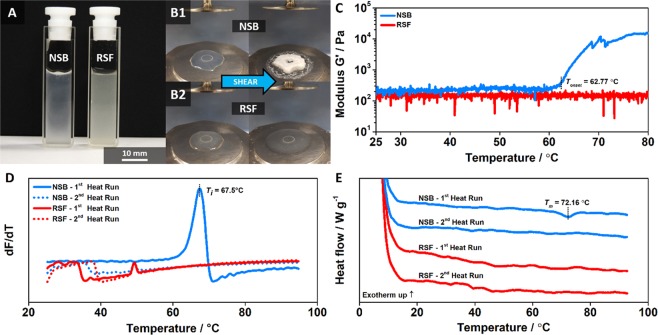
Rheological and thermo-analytical evaluation of RSF and NSB feedstock. (**A**) Prepared NSB and RSF solution (**B1**) Shear induced sample gelation of NSB, before and after rheological investigation, illustrating a visible change from a transparent solution to an opaque solid gel. (**B2**) RSF remained in a transparent and fluidic state, before and after rheological tests. (**C**) Constant oscillatory-temperature ramp curves of NSB and RSF feedstock. (**D**) DSF fluorescence signals of NSB and RSF feedstock, demonstrating the first derivative responses of two successive heating ramps. (**E**) Representative DSC thermograms of NSB and RSF feedstock. The curves were shifted vertically for clarity.

Rheological investigations (Supplementary Information: Fig. S3) show that NSB behaved like a weak gel with viscoelastic characteristics comparable to native silk fibroin (NSF), while RSF behaved overall like a typical liquid^5,13^. NSB and RSF featured apparent shear viscosities (viscosity measured at a constant shear rate of 1 1/s) of 1.84 ± 0.56  Pa·s at 6.4 ± 0.2% w/w and 0.15 ± 0.10  Pa·s at 5.1 ± 0.1% w/w, respectively, which is orders of magnitude lower than the mean average of 1722 Pa·s for NSF^5,13^. Importantly, despite this unnaturally low viscosity, gelation of NSB could be initiated purely by shear, occurring at a critical shear rate of 8.6 ± 4.4 1/s and shear stress of 9.8 ± 4.7 Pa. Our RSFs, produced to standard protocol^5^, never showed any shear-induced sample gelation, in accordance with previous studies^5^. Notably, the gelation of NSB was accompanied by a visible change from a translucent solution to an opaque solid gel (Fig. 3B1), with RSF remaining in a translucent and fluidic state (Fig. 3B2). Nozzle extrusion experiments confirmed the shear-induced sample gelation of NSB, demonstrating a clear change in appearance and rheological behaviour (Supplementary Information: Movie S1). Here we note that shear-induced gelation is generally accepted as an essential molecular self-assembly feature of natural silks that has independently evolved in moths and spiders to efficiently produce fibres and threads^4^. Our results strongly suggest that with this signature behaviour, NSB is also likely to retain this essential capability, so far only shown in natural silks in their native protein conformation, which is absent in standard RSFs^5,13^. In this regard, recent research work has shown that the reconstitution processing of silk fibroin disrupts the natural self-assembly capabilities leading to the loss of fibrillar formation through shear forces^14^. While the gelation of RSFs can be initiated by the application of various other external physical stresses or chemical compounds (e.g. temperature, sonication, strong acids, inorganic salts or organic solvents), these results demonstrated unoriented protein aggregation (or gelling), rather than an oriented protein fibrillation^15^.

To further elucidate the state of protein conformation within the NSB solution, we deployed a range of thermo-analytical techniques^16–22^. The thermal responses of NSB and RSF were analysed in a series of calorimetric, fluorometric and rheological tests (Fig. 3C–E). Briefly, differential scanning calorimetry (DSC) thermograms of NSB featured endothermic transition peaks at *T*_*m*_ = 72.2 ± 0.2 °C, which were characterized with denaturation enthalpies of *∆H* = 1.49 ± 0.39 J/g (1^st^ heat run). No transition peaks were identifiable upon reheating (2^nd^ heat run), demonstrating irreversible denaturation characteristics^16–18^. Thus, the DSC data presents a clear folding/unfolding transition, in which the heat input leads to a conformational change of the protein structure. These results agree well with previous DSC work on NSF, which presented similar transition peaks at *T*_*m*_ = 67.8–68.6 °C and denaturation enthalpies of *∆H* = 0.25–0.42 J/g^16–18^. Notably, DSC curves of RSF feedstock lacked any thermal response during both successive thermal runs. Comparably, differential scanning fluorimetry (DSF) highlighted similar temperature dependent denaturation transitions by probing the interactions of the protein feedstocks with a hydrophobic domain binding Sypro Orange fluorescent dye^19,20^. NSB featured a distinctive denaturation peak at *T*_*i*_ = 67.5 °C (1^st^ heat run), absent upon reheating (2^nd^ heat run), presenting identical signal characteristics to NSF^19,20^. To emphasise, the DSF results verify that the NSB solution contains folded fibroin proteins. During the thermal unfolding transition, the utilised dye binds to newly exposed hydrophobic groups within the protein core, which is reflected in the strong fluorescent transition peaks^19,20^. Conversely, RSF feedstock lacked a strong transition peak during both heating runs, displaying similar results to denatured NSB (2^nd^ heat run). Regarding protein secondary structures, it is believed that the NSB solution consists predominantly of disordered structures similar to NSF. However, the formation of small scale β-sheet structures during the extraction and processing steps, cannot be fully excluded and should be examined in a future study focussing on the details of the transitions^23^. Nevertheless, DSC and DSF data provide good reassurances of native like disordered structures, due to similarities in *T*_*m*_ and *T*_*i*_ results, previously only observed in native-silk solutions^16–20^. Both DSC and DSF observations of NSB and RSF were reaffirmed via thermo-rheology. Constant oscillatory-temperature ramp curves of NSB demonstrated that the elastic modulus *G’* was temperature independent until 55 °C, but increased rapidly at an onset temperature of *T*_*onset*_ = 62.8 ± 2.7 °C, linked to the structural phase change (liquid to solid) of the NSB^21,22^. These results agree well with previous studies on NSF, which highlighted thermal stability up to 55 °C, a lower temperature that we associate with the lower water content of NSF (at ~75% w/w)^21,22^. Importantly, our RSF presented no thermal response over the temperature ramp and stayed transparent, strongly supporting the argument that the proteins in the RSF are irreversibly denatured. All in all, the thermo-analytical data strongly suggests that NSB retains critical self-assembly characteristics and responses of NSF, and is a true aquamelt despite the process of extraction and purification. This retention of critical properties for biomimetic fibre assembly, underlines the key advantage for small bulk scale production of our NSB dope over traditional methods of making liquid silk feedstocks.

**Figure 4:**
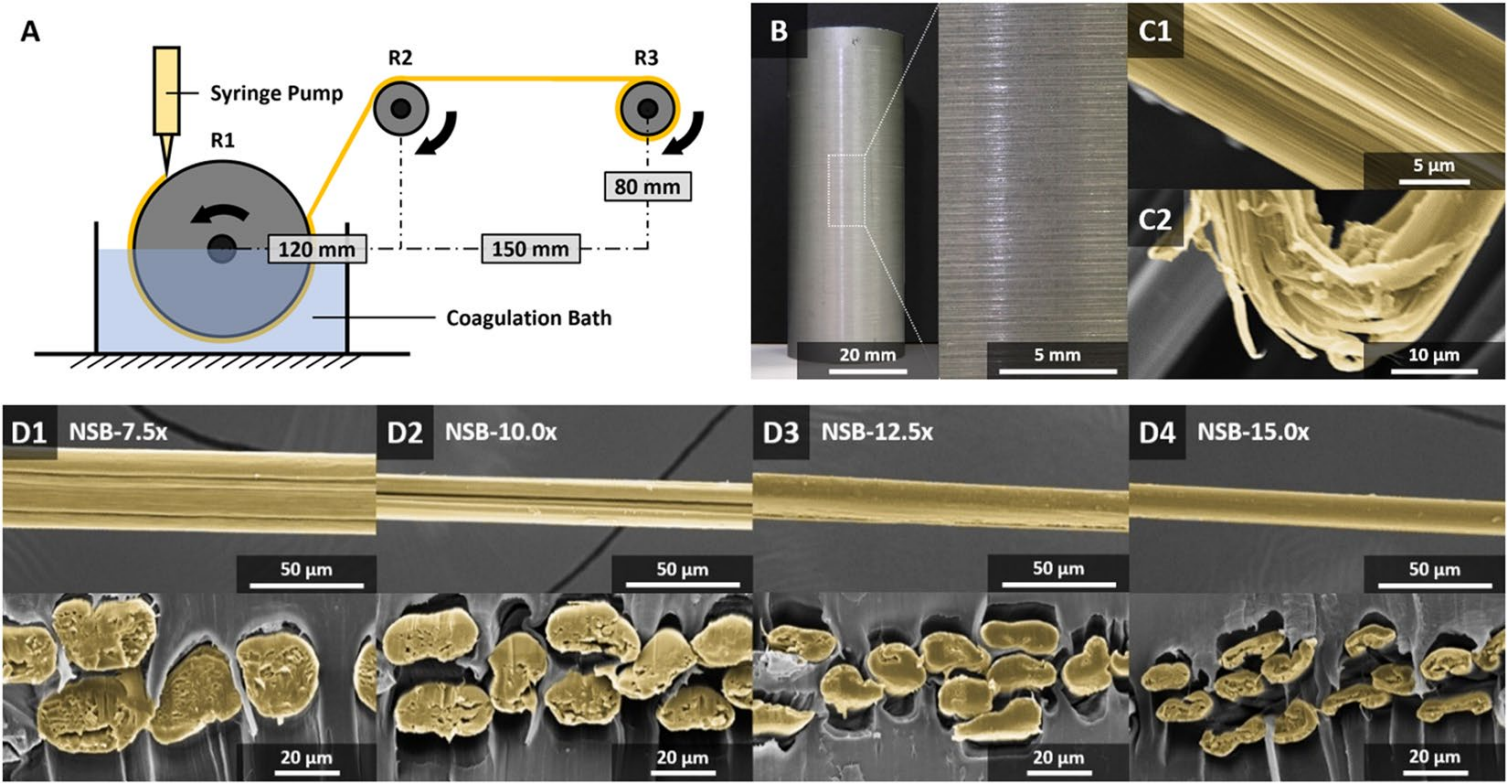
Wet spinning setup and morphologies of NSB fibres. (**A**) Custom designed wet-spinning setup. (**B**) Continuous spun NSB fibre on a cylindrical spool. (**C1–2**) SEM micrographs of NSB fibre samples, featuring nanofibrillar structures (colour enhanced). (**D1–4**) SEM micrographs of the longitudinal and transverse microstructure of NSB fibre samples (colour enhanced).

We move on from the feedstock to spinning fibres. In Nature, silk fibres with their impressive mechanical properties are spun via a purely water-based self-assembly system^4,6^. In contrast, the spinning of artificial ‘silk’ filaments generally relies on complex, toxic and energy intensive processes that nevertheless produces fibres that tend to be substandard when compared to native silk^6^. Thus, most artificial spinning approaches have individual challenges to overcome in aspects of feedstock/dope, fibre spinning and post-processing. Note that in relation to artificial we use ‘silk’ in quotation as judgement is still outstanding as to what constitutes a silk fibre as opposed to a biopolymer thread. Recent research has shifted into biomimetic spinning approaches, mimicking the native spinning process under controlled, mild & environmentally friendly conditions^24–33^. In particular recombinant fibres demonstrated greater improvements due to higher quality feedstocks, represented by higher molecular weights^6,34–36^. Still, most artificial spinning techniques used regenerated or recombinant silk feedstock/dope, which lacks native-like, phase transition capabilities^4,6^. To the best of our knowledge, so far no engineered i.e. synthetic or semi-synthetic silk feedstock/dopes that had native-like properties have been tested and published. In our case, using only a combination of moderate pH change and extensional stress (that leads to molecular alignment and water expulsion from the dope) continuous fibres were spun using NSB in a wet spinning system at ambient temperature (Fig. 4A,B). Remarkably, we could easily produce 1000 m of fibres at speeds up to 90 mm/s using only 2.5 ml of low protein mass NSB (6.4 ± 0.2% w/w) (Supplementary Information: Table S1). We note that, in contrast, the spinning of artificial ‘silk’ threads (be it from recombinant or reconstituted sources) typically requires mean protein masses over 15% w/w, with a few exceptions (Supplementary Information: Fig. S4). In our wet spinning setup, the NSB was extruded through a needle directly onto the surface of a rotating wetted roller, before pulling it through an aqueous PEG-based coagulation bath (pH 5.3 at 20 °C). This process facilitated the formation of a continuous proto-fibre, which was then further drawn in air between sequentially rotating rollers. To examine the effect of post-spinning draw-down, four draw-ratios i.e. 7.5x, 10x, 12.5x and 15x were imposed on our NSB filaments (NSB-7.5x, NSB-10x, NSB-12.5x, NSB-15x).

Importantly for the comparison to their natural NSF models, NSB fibres also featured hierarchically organized nanofibrillar structures (Fig. 4C1,2). This is significant, not only as it underlines the self-assembling feature of the NSB dope, but also because it is this specific trait that confers to natural silk fibres outstanding strength, extensibility and toughness^37^.

**Figure 5:**
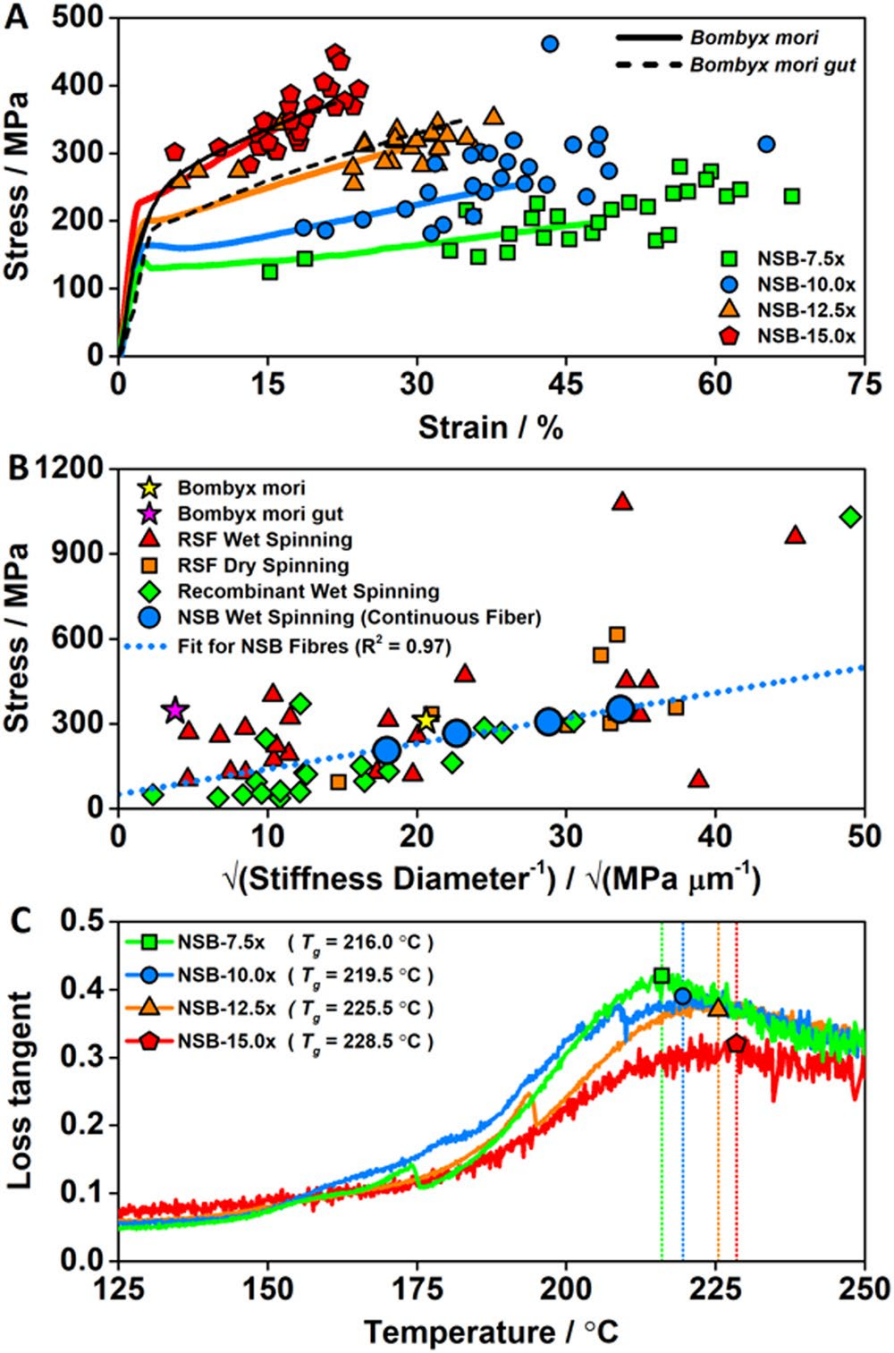
Mechanical properties of NSB fibres. (**A**) Representative quasi-static tensile stress–strain curves of NSB fibres fabricated at various draw-ratios, in comparison to naturally spun silk fibres and silk guts produced directly from the silk gland^2,45^. Scatter symbols give the breaking points of all specimens tested from each draw-ratio. (**B**) Comparison of the fracture-strength relationship of NSB fibres with naturally spun silk fibres, silk guts and various artificially spun silk fibres taken from the literature (Table 1, Supplementary Information: Table S2)^6,42^. (**C**) Representative loss tangent-temperature curves of NSB fibres.

We found that fibre gross morphologies displayed a direct relationship to the draw-ratio. Longitudinal directions featured smooth surfaces, but cross-section morphologies ranged from large crescent-like to small ribbon-like shapes, with areas from 585.6 ± 117.7 µm^2^ to 101.9 ± 20.2 µm^2^ for draw-ratios of 7.5x to 15.0x (Fig. 4D1–4), respectively. In addition, the relatively high varieties in cross-section morphologies are artefacts from the spinning set-up (roller/coagulation bath set-up) and depend on the processing parameters (e.g. flow rate of the syringe pump and draw-ratio). Overall, our NSB fibres exhibited excellent mechanical properties, comparable or exceeding natural *Bombyx mori* cocoon silks and silk guts produced directly from silk glands (Fig. 5A, Table 1). Importantly, this mechanical performance was achieved without post-processing steps and simply relied on the mechanical post-draw between successive rollers, effecting molecular structure by gently stretching the fibre^6^. Comparable post-draw techniques were used in previous studies^38–40^, however, the majority of studies relied on various post-processing treatments to alter the mechanical properties (e.g. to enhance the *β*-sheet content) of as-spun silk fibres, such as post-spin stretching in various aqueous or organic solutions (isopropanol, ethanol, methanol, ammonium sulphate, etc.), with or without heat input^6,35,36,41^. In this respect, by only varying the draw-ratio from 7.5x to 15.0x, *β*-sheet content increased gradually from 29 ± 2% to 36 ± 1% (Table 1, Supplementary Information: Fig. S5) as evaluated via Fourier-transform infrared spectroscopy. As a result, the progressive increase of the draw-ratio from 7.5x to 15.0x lead to an increase of the tensile Young’s modulus by 59% from 8.7 ± 1.2 GPa to 13.8 ± 1.4 GPa, and an increase of the ultimate tensile strength by 72% from 204.3 ± 40.7 MPa to 350.4 ± 41.3 MPa (Table 1). Contrarily, the elongation at break and toughness decreased 62% from 46.7 ± 12.5% to 17.6 ± 4.1% and 27% from 78.2 ± 30.2 MJ/m^3^ to 49.1 ± 15.5 MJ/m^3^, respectively. Notably, all fibres were characterized with distinctive yield points. In respect to reproducibility, the fabrication of thinner fibres appeared to be linked to increased fibre stiffness, with an increased consistency in fracture strength and extension properties (Figs 5A, S6)^6,42^. These fibre characteristics indicated that greater post-draw ratios are associated to higher degrees of crystallinity (and possibly improved molecular alignment), and vice versa, overall effecting the mechanical performance & consistency of the artificially spun silk fibres^6^.

**Table 1:** Cross-sectional areas, structure and quasi-static tensile properties of continuously spun NSB fibres, in comparison to natural silk fibres and silk guts.

Sample code	Cross section area^a^/µm^2^	*β*-sheet content^b^/%	Young’s modulus^c^/GPa	UTS*^c^/MPa	Strain at break^c^/%	Toughness^c^/MJ m^−3^
*Bombyx mori* ^*d* ^ ^2, 3^	~309	/	~8.5	~360.0	~18.5	~50.5
*Bombyx mori gut* ^*e *^ ^45^	~180000	/	~7.0	~346.0	~34.0	~80.0
NSB-7.5x	586 ± 118	29 ± 2	8.7 ± 1.2	204.3 ± 40.7	46.7 ± 12.5	78.2 ± 30.2
NSB-10.0x	311 ± 56	33 ± 2	10.2 ± 2.4	265.9 ± 59.7	38.0 ± 9.6	79.4 ± 39.5
NSB-12.5x	186 ± 31	35 ± 2	12.7 ± 2.2	306.8 ± 26.2	27.0 ± 7.9	65.3 ± 20.7
NSB-15.0x	102 ± 20	36 ± 1	13.8 ± 1.4	350.4 ± 41.3	17.6 ± 4.1	49.1 ± 15.5

^a^*n* = 120; ^b^*n* = 3; ^c^*n* = 28; ^d^Naturally spun silk fibres, un-degummed; ^e^Silk gut produced directly from the silk gland, un-degummed; *Ultimate tensile strength.

The fracture-strength relationship of the NSB fibre presented a similar trendline fit to other artificially spun silk fibres, indicating comparable energy release rates (Fig. 5B, Supplementary Information: Table S2)^6,42^. As previous research work demonstrated, natural silks tend to follow a fit with higher slope, representing a significant difference in their mechanical and structural characteristics^6,42^. Interestingly, naturally spun *Bombyx mori* silk (un-degummed) demonstrated relatively similar fracture-strength relationship values to NSB fibres spun at low draw-ratios, while silk guts presented completely different energy release rates characteristics, despite being spun from NSF. In this respect, our results demonstrated that the mechanical and structural characteristics of the spun fibres are not only dependent on the feedstock quality, but also on the spinning technique and processing parameters^6,42^. Forced reeled *Bombyx mori* silkworms showed similar connections between spinning and processing conditions, producing silk fibres with a wide spectrum of mechanical properties under controlled settings^2,3^. Thus, the fracture-strength relationship analysis indicated that the NSB spinning process has the potential to be further modified and optimized to produce fibres with further tuned properties.

For all silks, the structural and mechanical properties depend greatly on parameters and variables during the complete silk spinning process, which affect the molecular orientation and degree of order i.e. the ratio of crystalline to amorphous regions, as well as the formation and original morphology of the nanofibrils and their hierarchical organization^2,3^. We analysed structural variations brought about by the post-draw using dynamic mechanical thermal analysis (DMTA) (Fig. 5C, Supplementary Information: Fig. S7). An increased draw-ratio from 7.5x to 15.0x resulted in a significant shift of the glass transition temperature (defined via the loss tangent peak) from 216.0 °C to 228.5 °C, while correspondingly the loss tangent peak height decreased from 0.42 to 0.32. DMTA profiles indicated a clear decrease in molecular mobility for high draw-ratio fibres, suggesting a decrease in amorphous and disordered structures, representing an increase in crystallinity (*β*-sheet structure), which is in agreement with prior studies^2,43^. Additionally, thermal gravimetric analysis of low to high draw-ratio fibres supported these correlations (Supplementary Information: Fig. S8), with decreasing water content, associated with less amorphous regions, and higher decomposition temperatures characteristic of higher crystallinity (*β*-sheet structure)^2^. Overall, the thermal analysis of the NSB fibres via DMTA and TGA correlated with good agreement to the prior performed mechanical and spectroscopic investigations, indicating a clear relationship between the draw-ratio and the resulting silk protein structure within the artificially spun fibres.

## Conclusion

Summarizing our work, we propose that the process outlined here makes it possible to study a model aquamelt process systematically as it provides a gold standard dope for fundamental research and applied development. Further comprehensive studies are planned to evaluate the different aspects of the proteins’ structure within the NSB solution, alongside the proteins’ long-term stability over time. Beyond the ability to produce silk-like fibres with comparable qualities, our readily scalable, non-regenerated, aquamelt spinning dope presents a new and exciting low-energy process for forming novel silk-based structures, such as shear-induced microscale capsules and nanofibrils^23,37^. We anticipate that the research presented here will significantly advance understanding not only in how aquamelts are formed and spun, but also how their low-energy self-assembly could be applied in novel synthetic polymer processing.

## Materials and Methods

### Materials

*Bombyx mori* silkworms and cocoons were sourced from Italy and Kenya, respectively. Lithium bromide was acquired from Honeywell Fluka. Ammonia and PEG (*M*_*w*_ = 8000 g mol^−1^) were obtained from Fisher Scientific. Ammonium acetate, sodium carbonate, SYPRO orange dye and sodium carbonate were purchased from Sigma-Aldrich.

### Preparation of RSF and NSB feedstock

RSF feedstocks were prepared via an adapted standard method^5^. Briefly, pre-cut cocoons were boiled in a sodium carbonate solution (0.02 M) for 30 min, and then washed in type-I water for 20 min three times. The washed fibres were dried under vacuum at 50 °C until a constant weight was obtained. The dried fibres were dissolved in lithium bromide solution (9.3 M) at 60 °C for 4 h, before dialysis in cellulose acetate membranes (Medicell Membranes, UK; Mw = 12–14 kDa) against 2 L type-I water at 5 °C (type-I water was changed four times each day for 2 days). Conductivity readings were taken to confirm a conductivity of <5 µS at the final dialysis stage. Dialysed RSF was stored at 5 °C until further testing.

NSB feedstock was prepared using native silk glands from *Bombyx mori* silkworms (5^th^ instar, after final excretion) via a custom-developed dope extraction process. Briefly, the dope extraction process included an additive manufactured dope collector with wiring channels. The dope collector was inserted into a 50 mL glass beaker, filled with 20 mL of a 10 mM ammonium acetate extraction buffer solution (pH 7.3 at 10 °C; adjusted with a 1% v/v ammonia solution). Glands were dissected into a petri dish of type-I water at 5 °C, washed to remove detritus, and transferred to a second petri dish prepared as before. Cuts to the anterior and posterior middle gland were made, and the prepared gland placed on the wire of the dope collector (at the hairpin loop of the posterior middle and middle sections; 18 glands were used in total for each extraction process). Additional extraction buffer was added to cover the glands, and Parafilm placed over the top to prevent solvent loss. The filled dope collector was then stored at 5 °C overnight, allowing a gravity assisted extraction. The dope collector was removed from the beaker, resulting in a two-phase separated solution. The upper layer of buffered sericin was pipetted out, whilst the lower layer of buffered fibroin was filtered at 5 °C under gravity through a fine mesh to remove residual elements of gland epithelium. The filtered solution was then stored at 5 °C until further testing. An average of ~15 ml buffered fibroin and ~25 ml buffered sericin can be obtained per extraction process. Feedstock concentrations were determined by weighing of dry mass from known wet masses.

### Characterisation of RSF and NSB feedstock

SDS-PAGE was performed using fresh NSB, sericin and RSF solutions. Samples were diluted to 1% v/v and mixed with an equal quantity of pre-prepared sample buffer (Novex Tris-glycine SDS sample buffer) and reducing agent (NuPAGE Sample Reducing Agent), prepared to manufacturer’s instructions, giving a final working concentration of 0.5% v/v, for denaturing at 95 °C for 30 s. Samples, along with two protein standards, HiMark (Invitrogen) and Rainbow Marker (Amersham Full Range), were loaded on a Novex Tris-Glycine (4–12% gradient), and run at a constant voltage of 150 V with a max current of 40 mA. Total running time was 90 min. Gels were fixed for 30 min before Coomassie blue staining for 10 min. Gels were de-stained for 2 days before imaging with a BioRAD EZ Gel (Bio-Rad Laboratories, USA).

Rheological measurements were performed on a Bohlin Gemini rheometer (Malvern Instruments, UK), equipped with a temperature-control unit, a wetted environmental chamber and a steel CP4/40 cone-plate geometry (diameter: 40 mm; opening angle: 4°; truncation: 150 μm; closing speed: 0.1 mm s^−1^) for standard measurements (*n* = 10), and a CP4/20 cone-plate geometry (diameter: 20 mm, opening angle 4°; truncation: 150 µm; closing speed: 0.1 mm s^−1^) for heat ramp oscillatory measurements (*n* = 3). Samples were loaded with a 1000 µl Gilson pipettor, with the pipette tip trimmed to prevent excess shearing. Excess material around the cone was not removed and the sample area was not flooded with distilled water to prevent dilution. A pre-shearing stage (shear rate: 1 s^−1^; time: 100 s; temp.: 25 °C) was performed on all specimens, after an initial 300 s isothermal equilibration and the apparent shear viscosity (*η*_1_) was obtained by averaging the data from the final 30 s. Subsequent oscillatory (frequency: 100–0.1 Hz descending; strain amplitude: 0.02) and viscosity (shear rate: 0.04–250 s^−1^) experiments were performed at 25 °C, while separate modulus-increase (frequency: 10 Hz; stain amplitude: 0.02) experiments were performed via a temperature ramp of 1 °C min^−1^ from 25–80 °C.

Differential scanning calorimetry (DSC) measurements (*n* = 10) were performed on a Q2000 instrument (TA Instruments, USA), equipped with a refrigerated cooling system. DSC tests were performed from 5 to 95 °C with Tzero pans and hermetically sealed lids, followed by a second test cycle under the same conditions (sample weight range: 25 to 30 mg; heating rate: 5 °C min^−1^; purge gas: oxygen free nitrogen; gas flowrate: 50 cc min^−1^). The dry weight for each measured specimen was used to quantify the endothermic denaturation reaction.

Differential scanning fluorimetry (DSF) measurements (*n* = 15) were performed on a Rotor-Gene Q machine (Qiagen, NL) with SO (1:50 dilution) as the fluorescent probe (absorption maximum: 470 nm; emission maximum: 569 nm). The setup and procedure of the DSF were carried out as previously described^19,20^. Briefly, the instrument was set to spin the specimens (25 μL per sample; mix of diluted silk solution (2% w/v; 20 μL) and SO (5 μL)) at 400 rpm, while the fluorescence was measured continuously at 1 Hz with the temperature increasing from 25 to 95 °C at a rate of 0.4 °C min^−1^. Five independent fluorescence channels were measured, with the green signal channel (exciting: 470 nm; emitting: 510 nm) offering the best dynamic range for the fluorescence screening. All specimens were subjected to a second analysis cycle to confirm an irreversible denaturation.

### Preparation of RSF, NSB and sericin films

Films were produced by pipetting 400 µl of feedstock into the circular grooves of a 24-well cell plate lid and dried at room temperature until the weight of the final films were constant. Film samples were stored in a standard 50% relative humidity at ambient temperature until testing.

### Characterisation of RSF, NSB and sericin film

Fourier-transform infrared spectroscopy (FTIR) was performed on RSF, NSB and sericin film specimens at dry state via a Nicolet 6700 FTIR spectrometer (Thermo Scientific, USA), which was coupled to a Golden Gate single bounce diamond attenuated total reflectance (ATR) module (Specac Ltd, UK) and equipped with a liquid nitrogen cooled mercury-cadmium-telluride detector. Spectra acquisition was performed at a 4 cm^−1^ resolution from 650–4000 cm^−1^. The entire light-path of the instrument was continuously purged with dry air, eliminating spectral interference due to atmospheric water vapor. *Note:* FTIR spectroscopy was performed to detect & compare distinctive spectral features of the two main “known” proteins, fibroin and sericin, in film form and at dry state.

### Wet spinning of continuous NSB fibres

Artificial spun fibres were produced via a custom-made wet spinning system under different draw-down ratios at ambient temperature and RH (Table S1). Briefly, NSB feedstock was pumped via a Legato 100 Syringe Pump (KD Scientific, USA) through a plastic needle tip (material: PP; inner diameter: 0.38 mm) directly onto a first roller, which was half immersed in a coagulant bath (15% w/v PEG solution with 10 mM ammonium acetate; pH 5.3 at 20 °C, adjusted with acetic acid). Due to the roller’s rotation, the extruded feedstock formed a continuous fibre-shaped geometry before entering the coagulation bath. The semi-solid proto-fibre was than redirected by hand onto a second rotating roller, and lastly collected on a third rotating & linear moving roller. The resulting fibres were immersed in type 1 water overnight at 20 °C to remove coagulant (PEG deposits) from the fibre surfaces, dried and stored in a standard 50% relative humidity at ambient temperature until testing.

### Characterisation of NSB fibres

The morphological and cross-sectional analysis of all fibres were performed using a JCM-5000 NeoScope (Jeol, JPN) scanning electron microscope (SEM), at a high-vacuum and 10 kV. SEM sample preparation was performed as follow: NSB fibres (length: 2 m) were gently wound around a thin sheet of (low density) polyethylene, to produce a section of multiple orientated fibres. This section was placed in between two further thin sheets of (low density) polyethylene, and melted together, between two glass plates, with a small amount of pressure at ~100 °C (*Note:* No morphological alterations to the fibres are expected, due to brief exposure times far below decomposition temperatures, and low water content (<5%), as demonstrated by DMA and TGA, respectively). The embedded fibres were sectioned with a razor edge at a 90° angle into several small blocks. These blocks were placed fibre cross-section up and coated under an 18 mA current for 150 s in a SC7620 sputter coater (Quorum Technologies, UK) giving a 7 nm coating of gold/palladium. The image processing software ImageJ was used to perform morphological measurements.

Quasi-static tensile tests were executed on single NSB fibre specimens (*n* = 28, gauge length: 10 mm) in cardboard frames by using a zwickiLine Z0.5 (Zwick-Roell, GER) tensile testing machine and a 5 N load cell. Fibres were mounted under low tension and a strain rate of 20% min^−1^ was used until fracture (only specimens that broke in the middle were used) at ambient condition and 50% relative humidity. The average cross-sectional areas (*n* = 120) were applied to the fibre specimens used in the tensile tests. *Note:* The “engineering” stress and strain values were utilised throughout this work.

FTIR spectroscopy was performed in ATR mode on randomly aligned NSB fibres (*n* = 3) for secondary structural analysis. Spectra acquisition was performed at a 4 cm^−1^ resolution from 650–4000 cm^−1^. The entire light-path of the instrument was continuously purged with dry air, eliminating spectral interference due to atmospheric water vapor. The deconvolution of amide I bands (1580–1720 cm^−1^) was carried out using OriginPro and a linear baseline correction was performed. The second derivatives of the absorption spectra were used to define the number and position (minima points) of peaks and fixed for the deconvolution process. The assignment of the amide I band peaks was performed based on previous research^44^: 1663–1696 cm^−1^ to *β*-turn conformation, 1647–1655 cm^−1^ to random coil and/or helical conformation, and 1622–1637 cm^−1^ to *β*-sheet conformation. A Gaussian function was selected for curve fitting^33^.

Dynamic mechanical thermal analysis (DMTA) tests were performed on a Q800 (TA Instruments, USA), equipped with a liquid nitrogen cooling system. Full range temperature experiments (−100 °C to 250 °C) were conducted on single NSB fibres (*n* = 3) under multi-frequency strain mode (temperature ramp rate: 3 °C min^−1^; frequency: 1 Hz; dynamic strain: 0.2%). A preload of 0.01 N was applied throughout the experiments.

Thermal gravimetric analysis (TGA) measurements were performed on a Q500 thermogravimetric analyser (TA Instruments, USA). NSB fibres of (*n* = 3) approx. 0.3–1.0 mg were transferred into pre-tared aluminium pans and heated at a rate of 3 °C min^−1^ from ambient temperature to 300 °C in nitrogen gas (flow rate: 60 cc min^−1^).

### Movie S1 - Shear-induced gelation test of RSF and NSB feedstock

A Legato 100 Syringe Pump (KD Scientific, USA) was used to extrude RSF and NSB spinning dope through a plastic needle tip (material: PP; inner diameter: 0.38 mm, Sorenson #28480) vertically down at a constant flow rate of 100 ml h^−1^ and room temperature.

## Supplementary information

Supplementary information

Supplementary Movie S1

## Data Availability

The dataset generated during and/or analysed during the current study is available in the Zenodo open-access repository - 10.5281/zenodo.3457387.
