# Supplementary material for: Artificial spinning of natural silk threads
Source: Sci Rep. 2019 Oct 28;9:15428. doi: 10.1038/s41598-019-51589-9 (PMC6817873; doi:10.1038/s41598-019-51589-9)
Supplement: Supplementary file 1 — Supplementary information [file 41598_2019_51589_MOESM1_ESM.docx]

Supplementary Information

Artificial spinning of natural silk threads

Martin Frydrych, Alexander Greenhalgh and Fritz Vollrath*

Dr. Martin Frydrych, Alexander Greenhalgh B.Sc. and Prof. Fritz Vollrath.

Department of Zoology, University of Oxford, Zoology Research and Administration Building, 11a Mansfield Road, Oxford, X1 3SZ, United Kingdom.
E-mail: [*fritz.vollrath@zoo.ox.ac.uk*](mailto:fritz.vollrath@zoo.ox.ac.uk)

**Supplementary Figures and Tables**


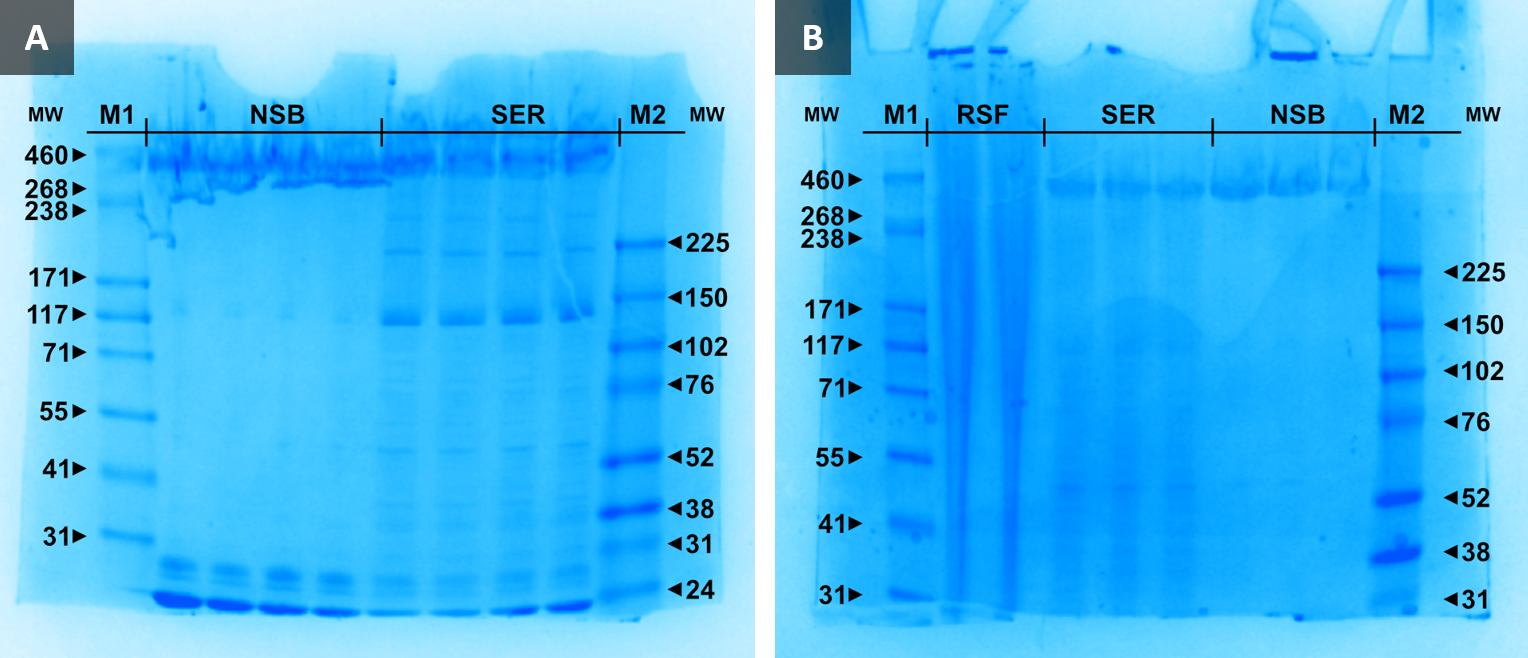


**Figure** **S1** SDS-PAGE analysis of (A) NSB and SER, (B) RSF, SER and NSB solution (MW: Molecular weight; M1: HiMark protein standard (31- 460 kDa); NSB: Extracted fibroin layer; SER: Extracted sericin layer; RSF: Regenerated silk fibroin; M2: Rainbow protein standard (24-225 kDa)). *Note:* SER and NSB samples in Figure 1B showed aggregation due to age (in week-old samples), and are not representative of fresh sample bands.


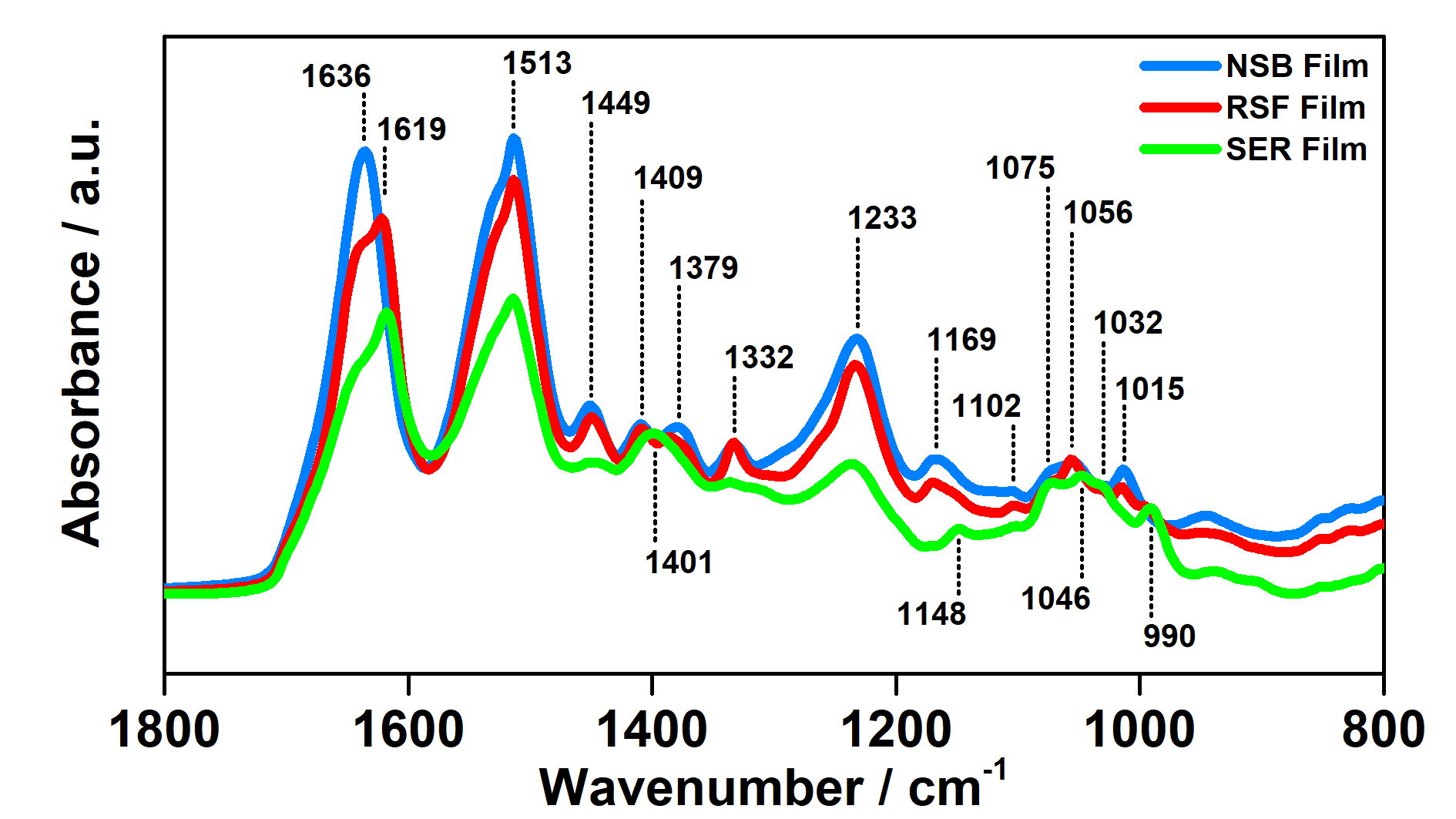


**Figure** **S2** Representative FTIR spectra of NSB, RSF and sericin (SER) films in the range of 1800-800 cm^-1^. (*Note:* FTIR spectroscopy was performed to detect & compare distinctive spectral features of the main “known” proteins, fibroin and sericin, in film form and at dry state). Briefly, NSB and RSF films were characterised by the Amide I and II bands (at 1636 cm^-1^, 1619 cm^-1^ and 1513 cm^-1^; corresponding to *β*-sheet structure), the bending vibration of CH_3_ or CH_2_ (at 1449 cm^-1^ (*β*-sheet structure), 1409 cm^-1^ and 1379 cm^-1^), the bending or wagging vibration of CH_2_ or CH (at 1332 cm^-1^ (*α*-helix structure)), the Amide III band (at 1233 cm^-1^; corresponding to random coil and *α*-helix structure), the stretching vibration of C-N, C-O or C-C (at 1169 cm^-1^, 1102 cm^-1^ (*α*-helix structure) and 1056 cm^-1^), and the rocking vibration of CH_2_ (at 1032 cm^-1^, 1015 cm^-1^ and 960 cm^-1^ (*β*-sheet structure)).^1^ In comparison, SER film presented absorption bands related to the sericin protein, characterised by the bending vibration of CH_2_ or OH (at 1401 cm^-1^), the stretching vibration of CH_2_, C-O, C-N or C-OH (at 1148 cm^-1^, 1075 cm^-1^ and 1046 cm^-1^), and the rocking vibration of CH_2_ (at 990 cm^-1^ (*β*-sheet structure)).^1^ The raised hydroxyl content contributed to a broad absorption peak at around 3400 cm^-1^ (data not shown), which can be linked to a greater presence of water.^1^ Overall, FTIR spectroscopy demonstrated: (I) The spectra curves of NSB and RSF films are relatively similar, indicating that the extracted NSB solution consists mainly of fibroin proteins. (II) The spectra curve of the SER film is relatively different to the NSB and RSF films, indicating that the extracted sericin solution consists mainly of sericin proteins.


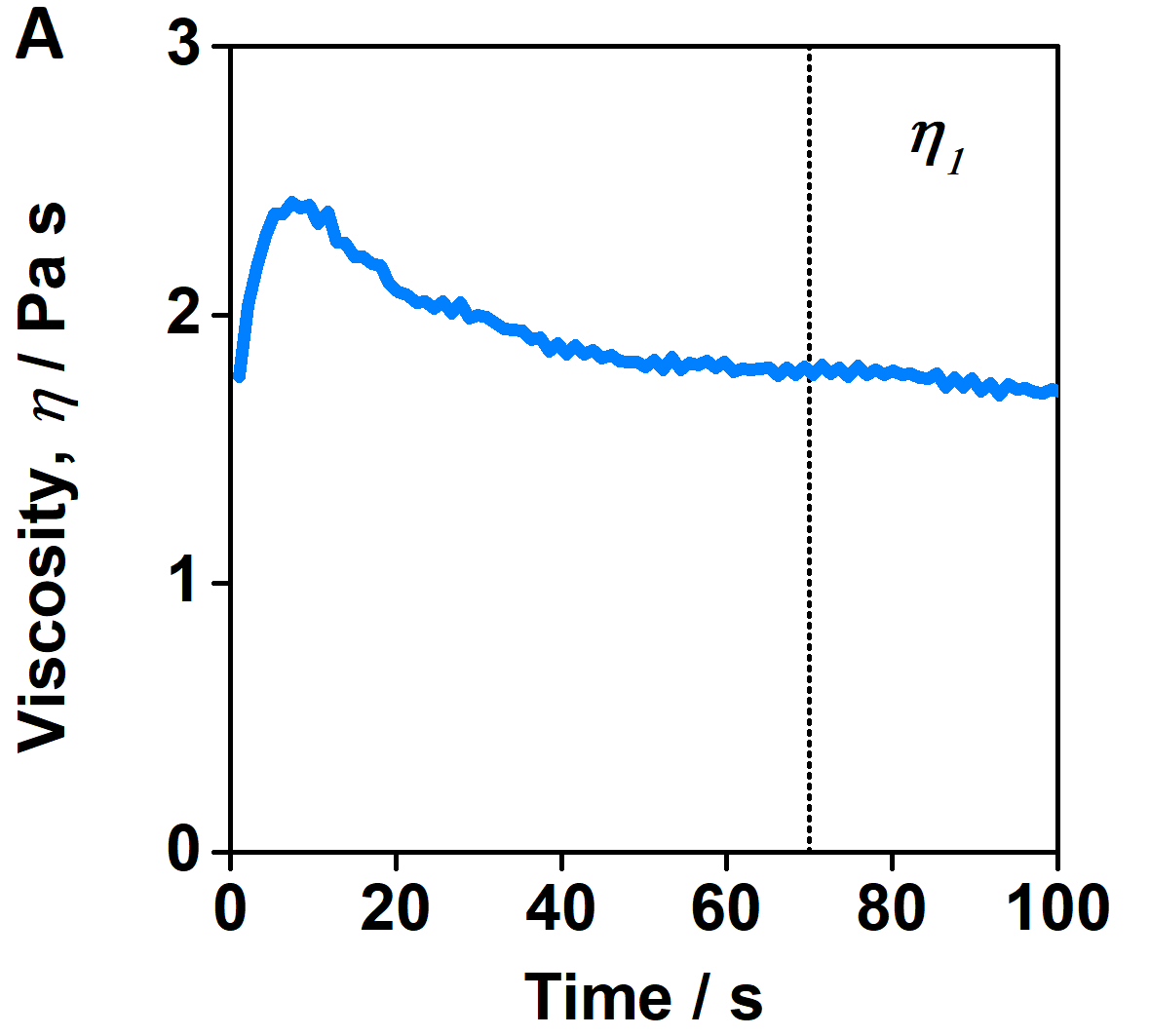

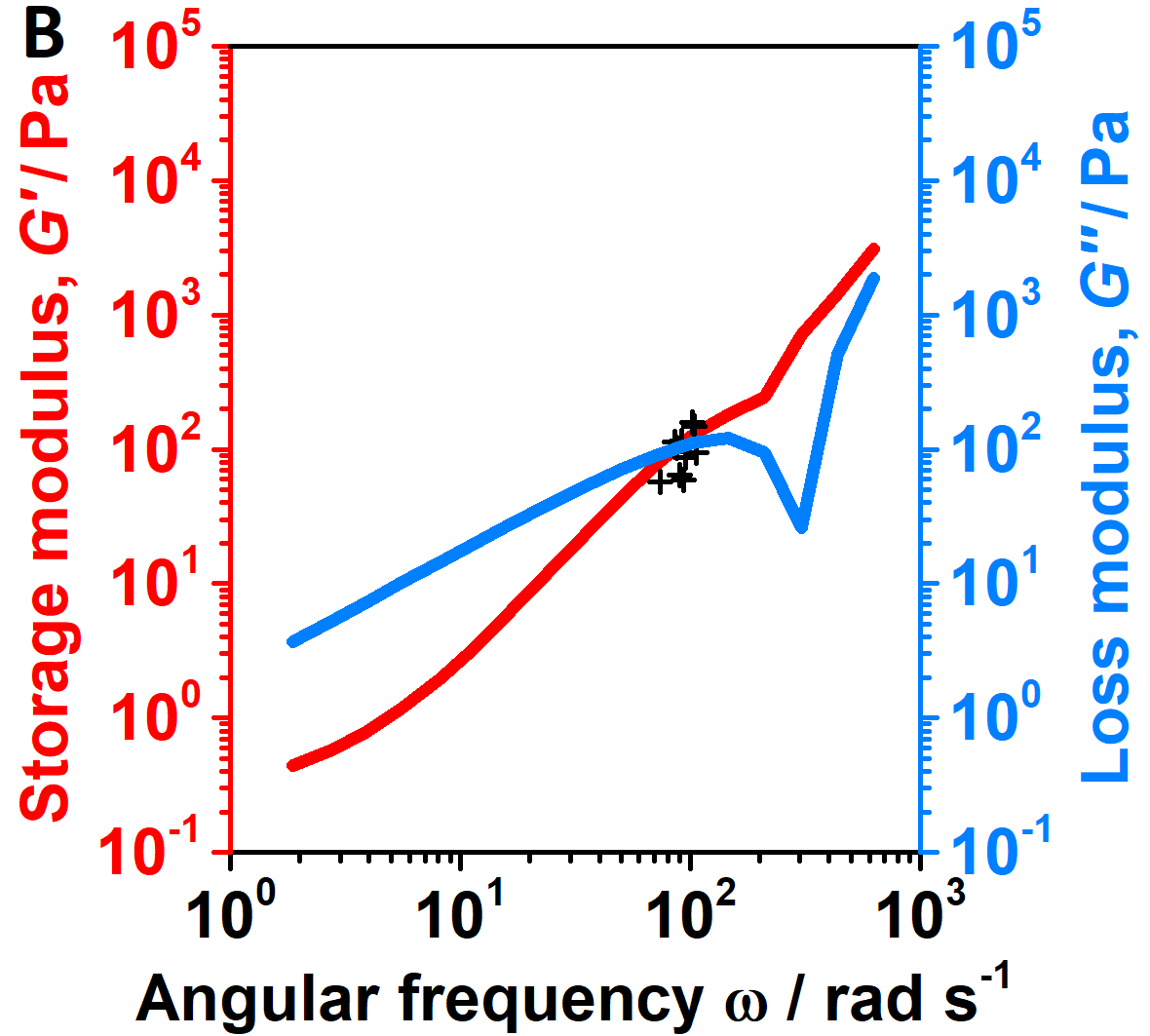

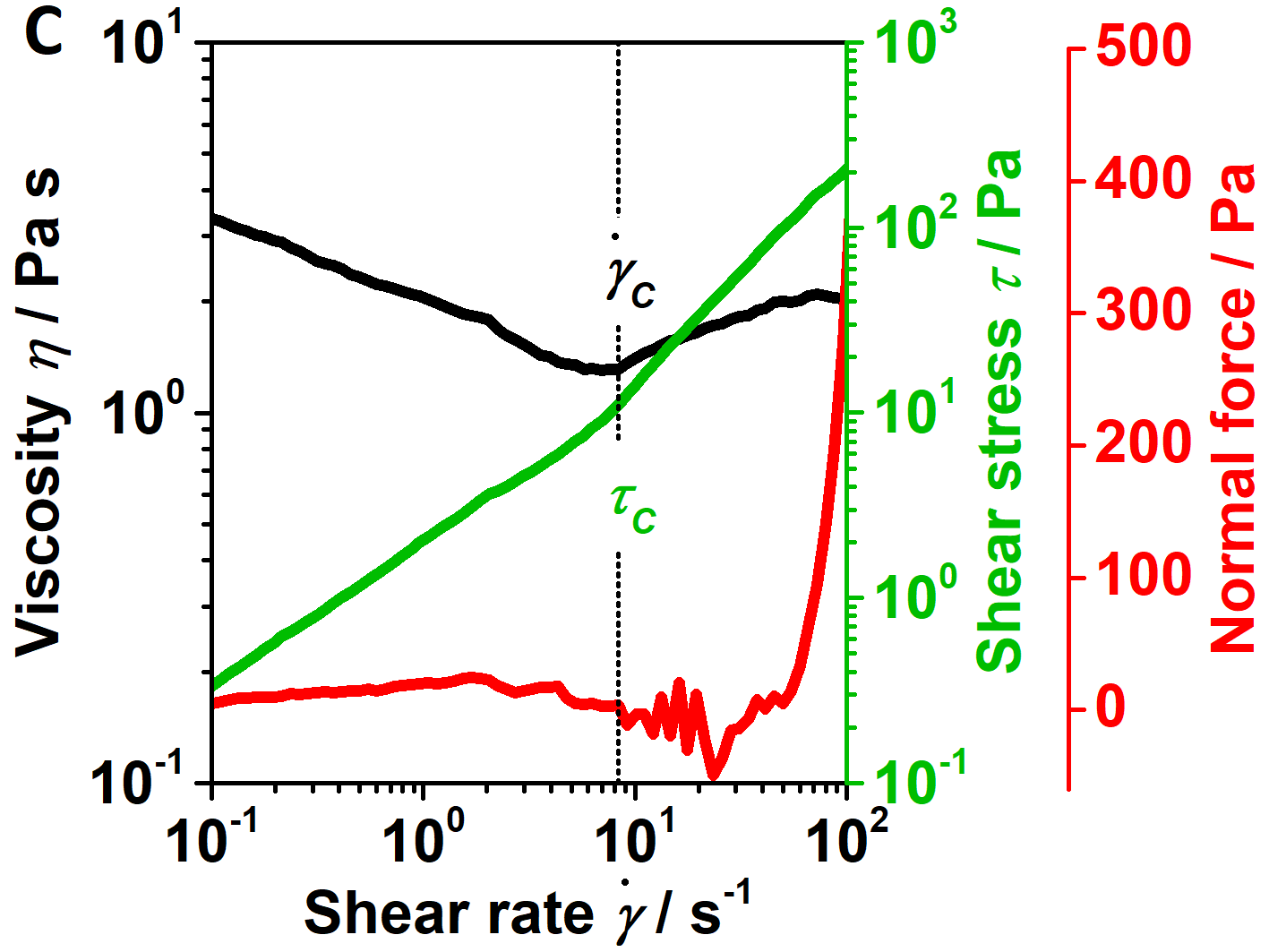


**Figure S3** (A) Representative apparent shear viscosity curve of NSB tested at a constant shear rate of 1 s^-1^ for 100 s. The apparent shear viscosity*, η_1_*, was obtained by averaging the final 30 s of data of each specimen.^2^ Briefly, NSB at a dry weight fraction of 6.41 ± 0.21% w/w featured an apparent shear viscosity of *η_1_* = 1.84 ± 0.56 Pa s. In comparison, native silk from *Bombyx mori* features a significantly higher apparent shear viscosity of *η_1_* = 1722 ± 935 Pa s, due to its high dry weight fraction of 24 ± 2.5% w/w.^3^ *Note:* RSF at a dry weight fraction of 5.10 ± 0.05% w/w was characterised with a *η_1_* = 0.15 ± 0.10 Pa s, overall demonstrating an order of magnitude difference to NSB. (B) Representative constant strain oscillatory sweep curves of NSB, presenting the elastic (*G’*, red) and viscous (*G’’*, blue) moduli against angular frequency. Frequency dependent crossover points (G′ = G″), representing the boundary between solid- and liquid-like viscoelastic behaviour, are plotted as black crosses. Briefly, NSB at a dry weight fraction of 6.41 ± 0.21% w/w demonstrated similar viscoelastic characteristics as native silk protein solutions and behaved like a weak gel.^3^ At higher angular frequencies the elastic modulus dominated (*G′* > *G″*), while at lower angular frequency the viscous modulus dominated (G′ < G″). NSB featured a crossover frequency of *ω_x_* = 93.17 ± 9.29 rad s^-1^ and a crossover modulus of *G_x_* = 99.68 ± 33.04 Pa. In comparison, native silk from *Bombyx mori* at a dry weight fraction of 24 ± 2.5% w/w features a significant lower crossover frequency of *ω_x_* = 5.6 ± 2.5 rad s^-1^ and a significant higher crossover modulus of *G_x_* = 3338 ± 666 Pa.^3^ *Note:* RSF at a dry weight fraction of 5.10 ± 0.05% w/w behaved like a liquid, demonstrated no frequency dependent crossover point and the viscous modulus *G″* dominated at all frequencies (G′ < G″), which is in accordance with previous studies.^4^ (C) Representative viscosity-shear rate curve with shear stress and normal force responses of NSB. Briefly, NSB at a dry weight fraction of 6.41 ± 0.21% w/w demonstrated shear thinning behaviour in the low shear rate regime (i.e., the viscosity decreases as the shear rate increases), while in the high shear rate regime the viscosity of NSB increased. This rheological effect is linked to the shear induced sample gelation and accompanied by an increase in shear stress, as well as a rapid change in normal force (lift-off effect).^5^ Previous studies demonstrated this phase change in native silk from *Bombyx mori* depends on its viscosity and can be initiated via the shear rate or shear stress.^6^ In this respect, the onset of the shear induced gelation in NSB feedstock occurs at the critical shear rate of = 8.63 ± 4.39 s^-1^ or at a critical shear stress of = 9.77 ± 4.70 Pa (values for and were determined by identifying significant slope changes in the related viscosity, shear stress and normal force curves). *Note:* The data in the high shear rate regime should be viewed with caution, due to the phase change from fluid to solid effecting rheological measurements. RSF at a dry weight fraction of 5.10 ± 0.05% w/w behaved like a Newtonian fluid and showed no shear induced sample gelation, which is in accordance with previous studies.^4^ It was characterised with a minimal shear thinning behaviour, a linear increase in shear stress and no change in normal force over the complete shear rate regime.


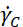

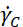

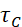

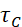

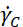

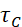

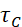


**Table S1** Wet spinning process parameters for NSB fibre fabrication.

| **Sample  code** | **Flow rate / ml h^-1^** | **Roller 1 / mm s^-1^** | **Roller 2 / mm s^-1^** | **Roller 3 / mm s^-1^** | **Roller Ratio** | | **Total draw-ratio** |
| --- | --- | --- | --- | --- | --- | --- | --- |
|  |  |  |  |  | 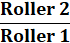 | 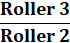 |  |
| NSB-7.5x | 0.75 | 2 | 5.33 | 15.00 | 2.67 | 2.81 | 7.5 |
| NSB-10.0x |  | 3 | 8.00 | 30.00 | 2.67 | 3.75 | 10 |
| NSB-12.5x |  | 4 | 10.67 | 50.00 | 2.67 | 4.69 | 12.5 |
| NSB-15.0x |  | 6 | 16.00 | 90.00 | 2.67 | 5.63 | 15 |


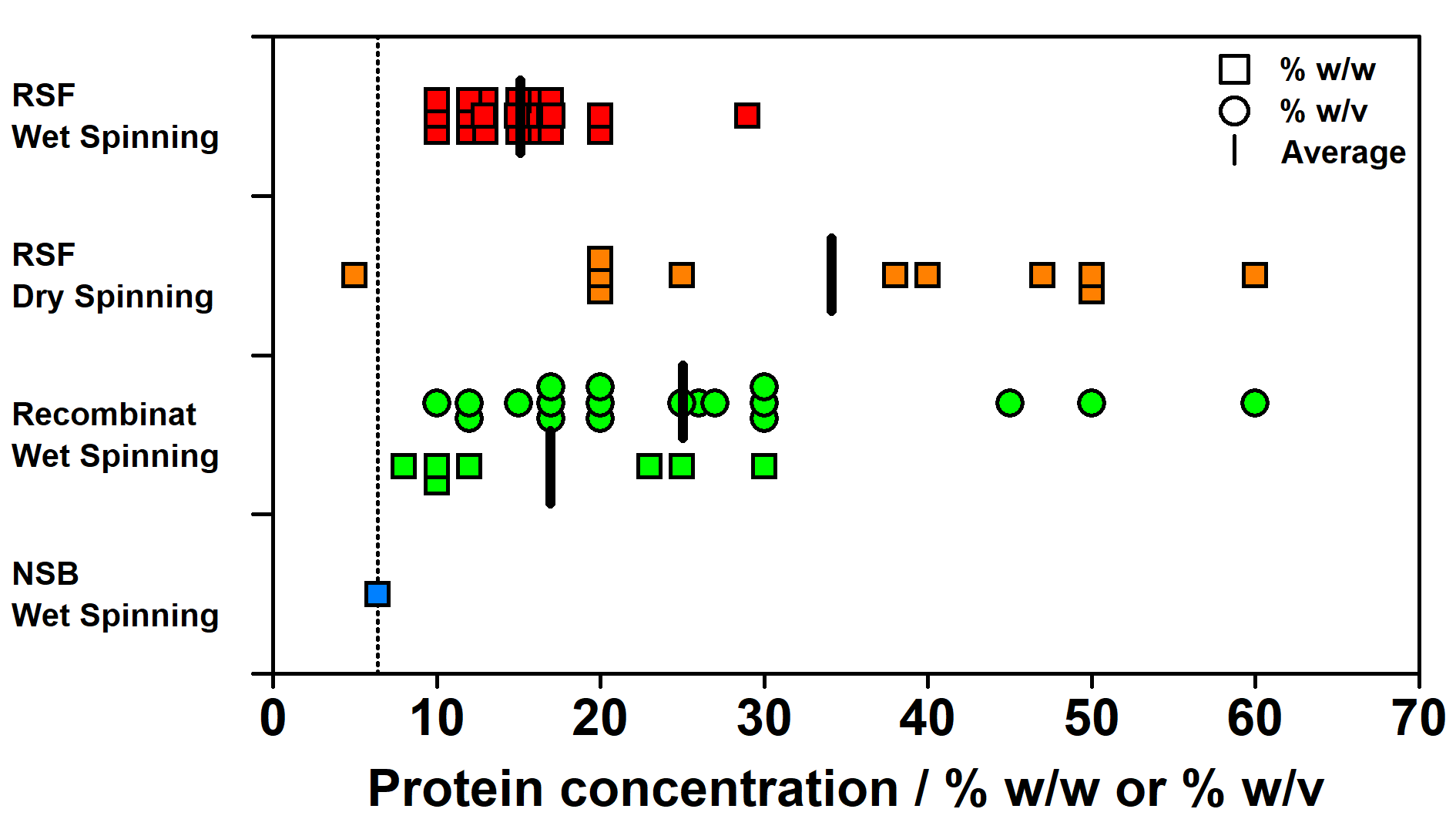


**Figure** **S4** Comparison of the protein concentration of NSB spinning dope with various spinning dopes from literature (*Note:* The fabrication processes of artificially spun silk fibres can be divided into wet or dry spinning. Data and references can be found in Table S2).^7,8^ Briefly, NSB spinning dope for wet spinning featured a protein concentration of 6.41 ± 0.21% w/w, presenting a significantly lower protein concentration then native silk dope from *Bombyx Mori* (24 ± 2.5% w/w).^3^ In comparison to NSB spinning dope, RSF and recombinant spinning dopes feature in general higher protein concentrations. For instance, spinning dopes for RSF wet spinning ranged from 10.0 to 29.0% w/w (mean of 15.1% w/w), while spinning dopes for RSF dry spinning ranged from 5.0 to 60.0% w/w (mean of 34.1% w/w). Recombinant spinning dope for wet spinning ranged from 10.0 to 30.0% w/w (mean of 15.1% w/w) or from 8.0 to 60.0% w/v (mean of 24.65% w/v).


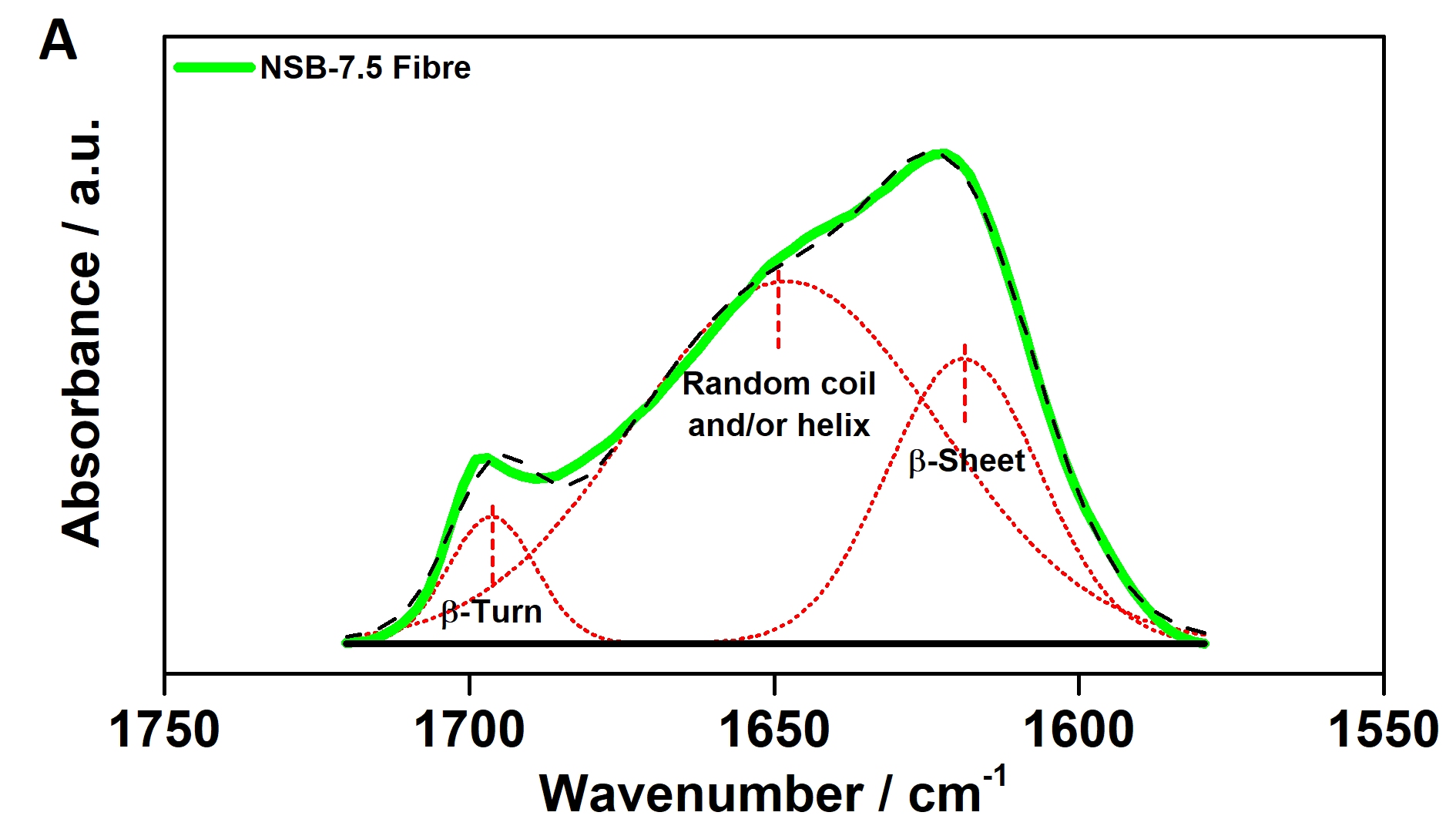

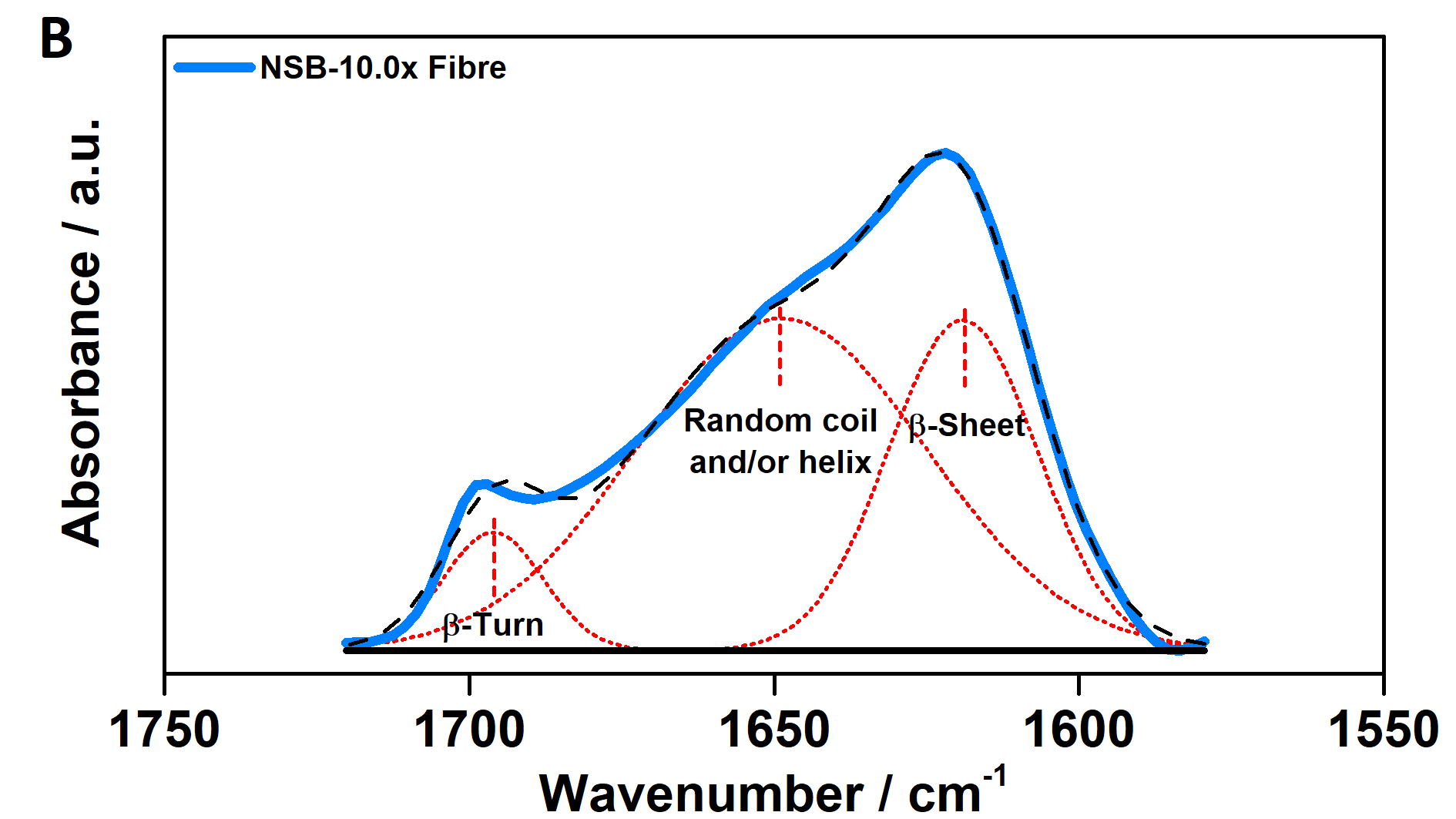

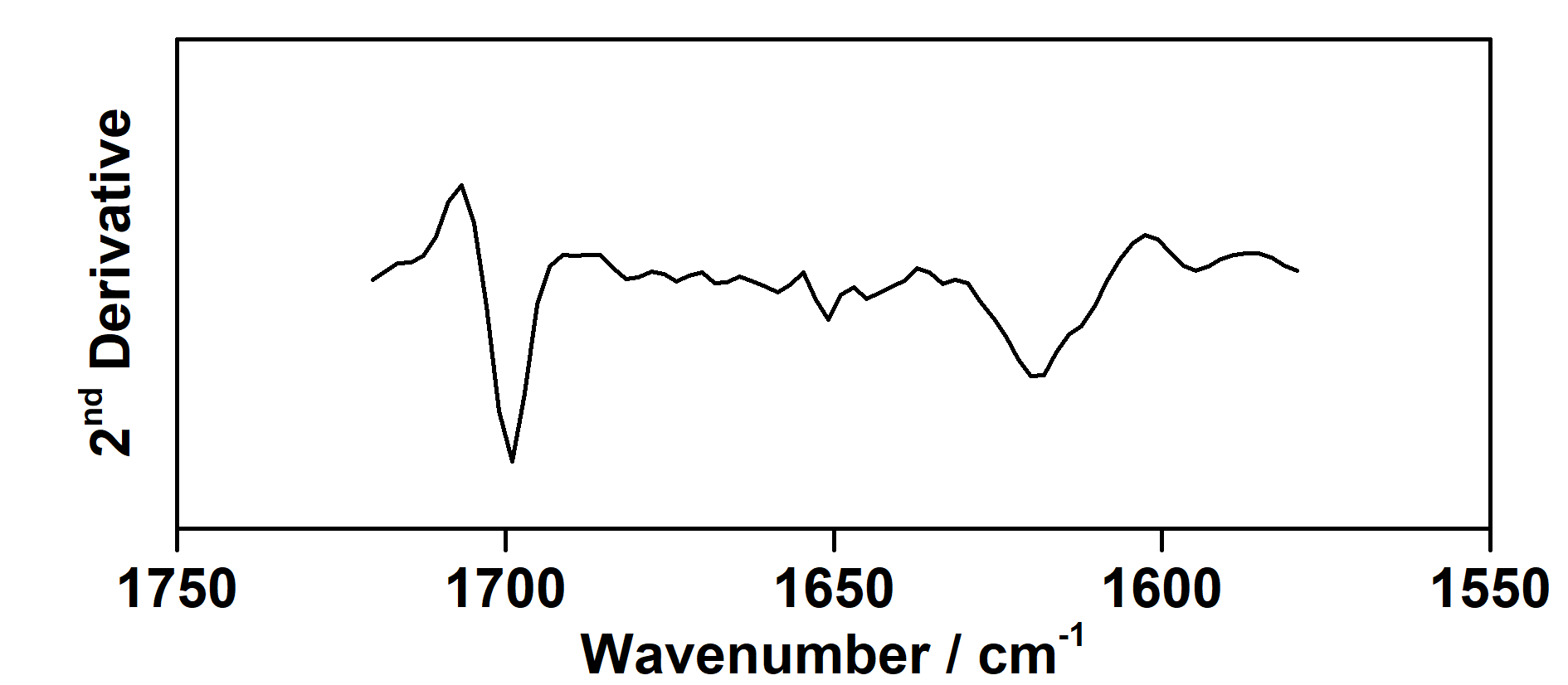

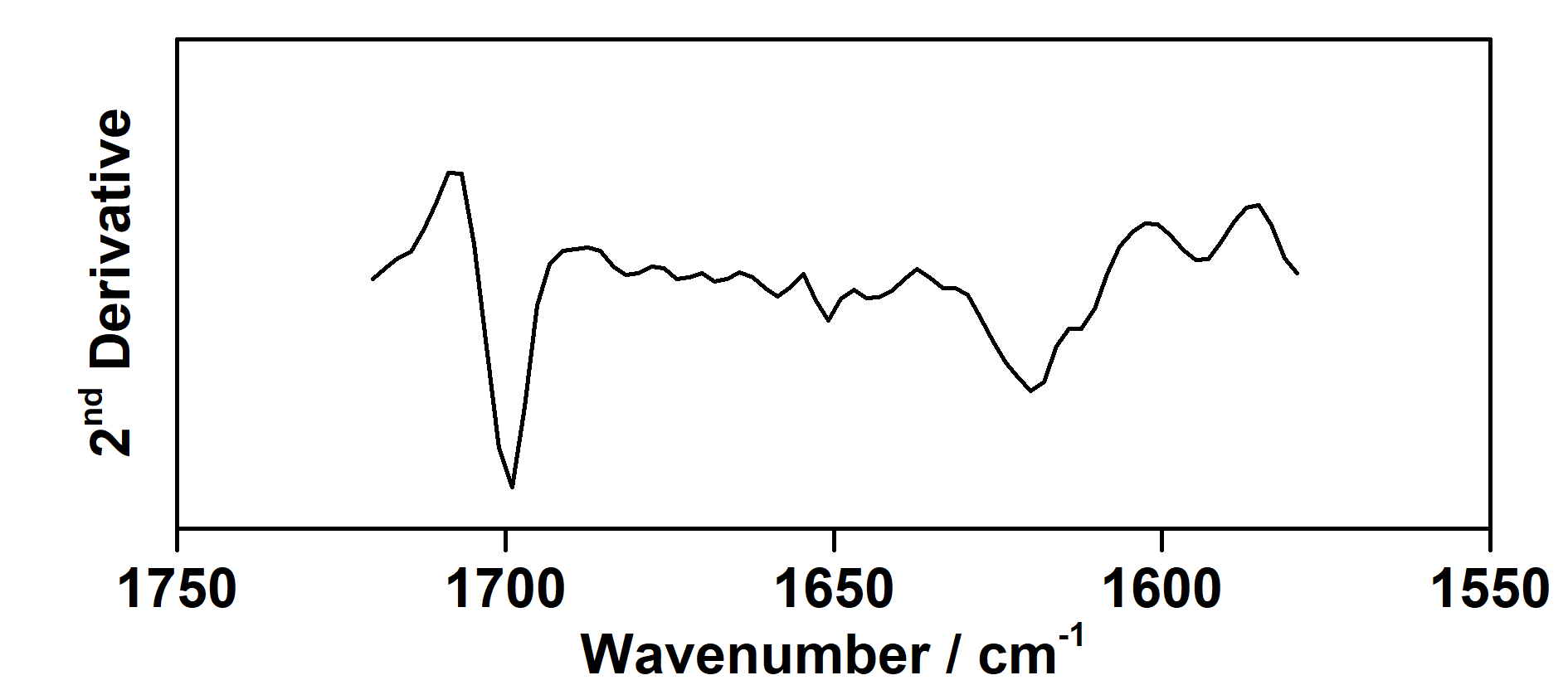

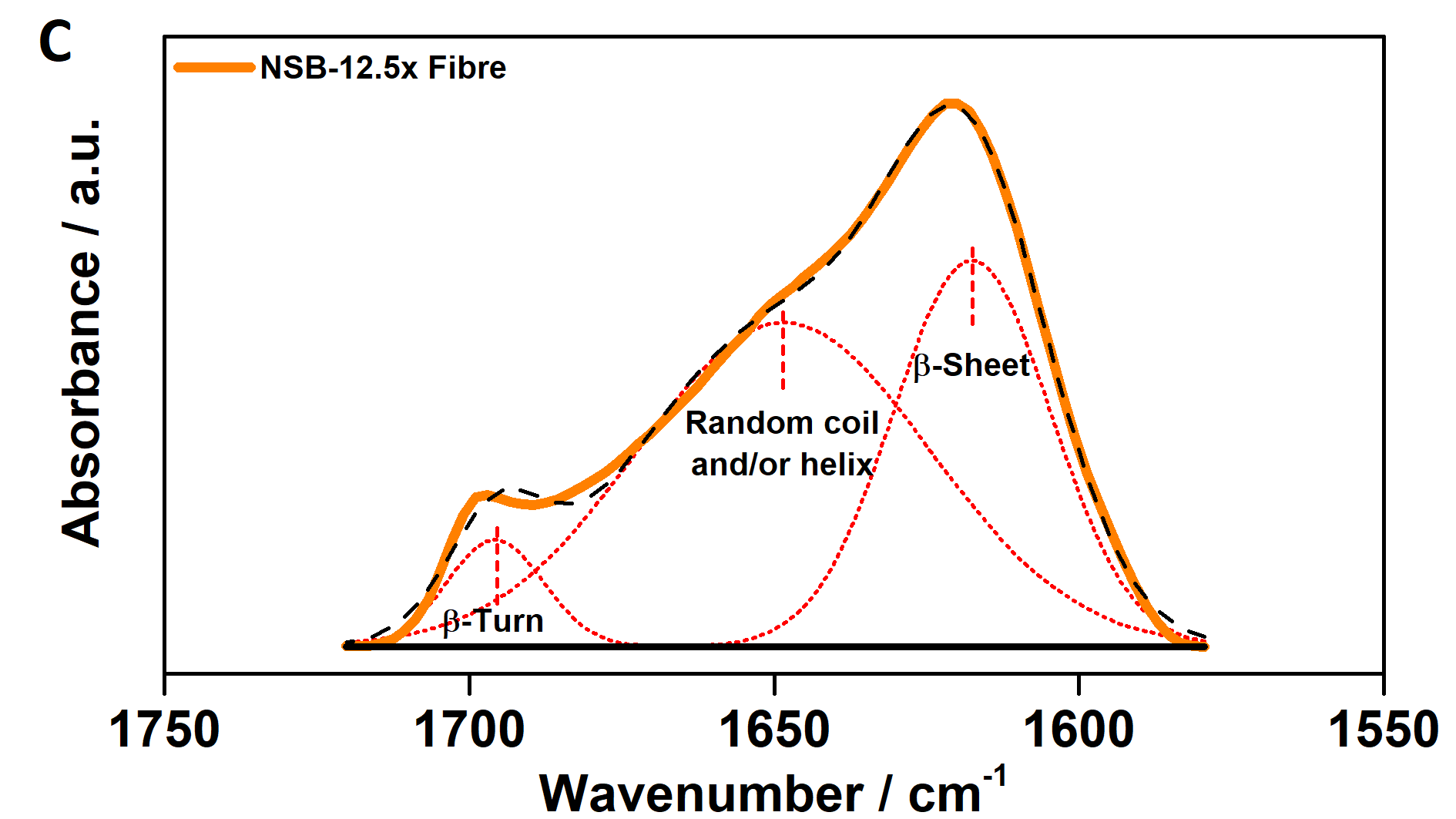

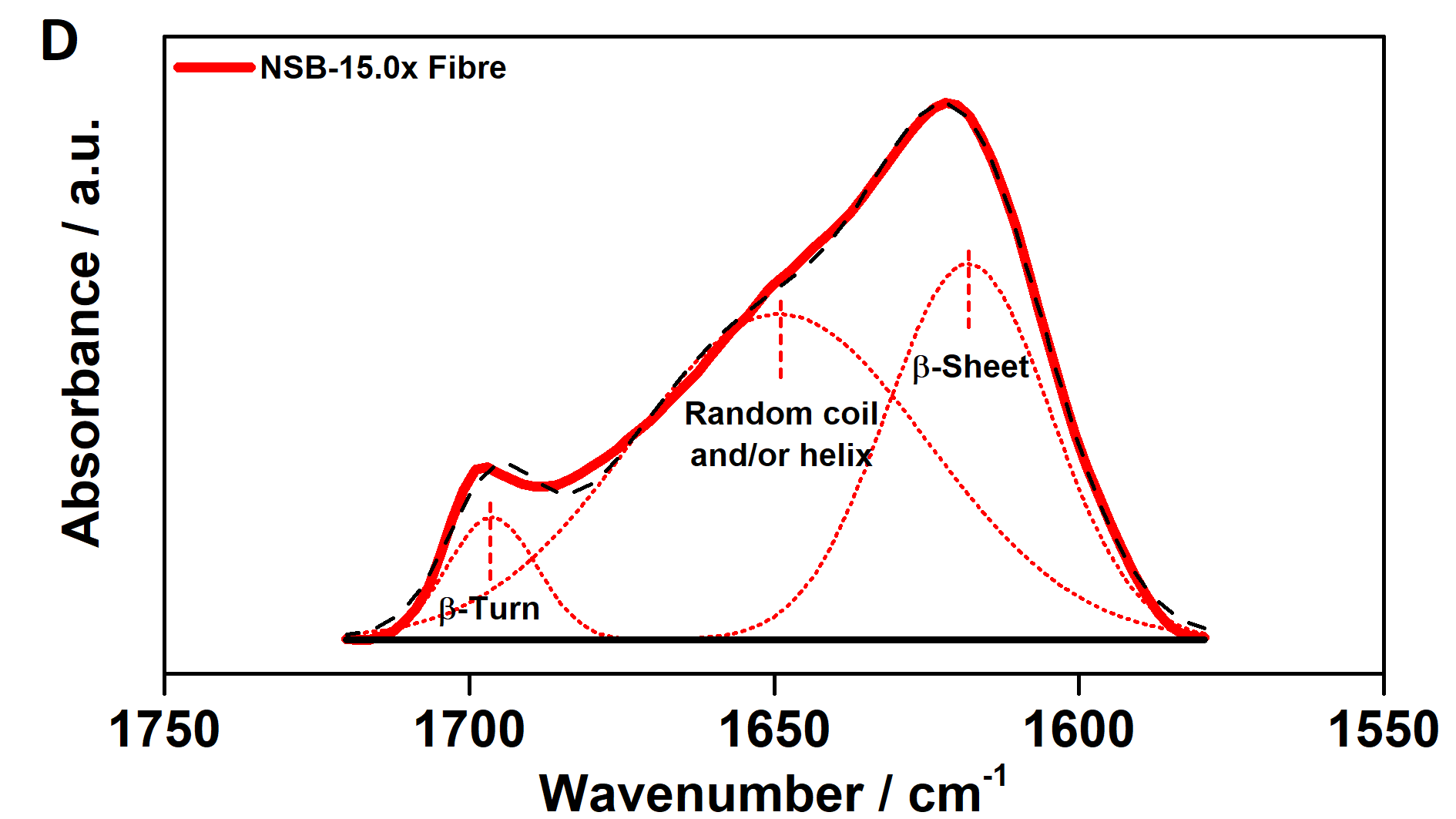

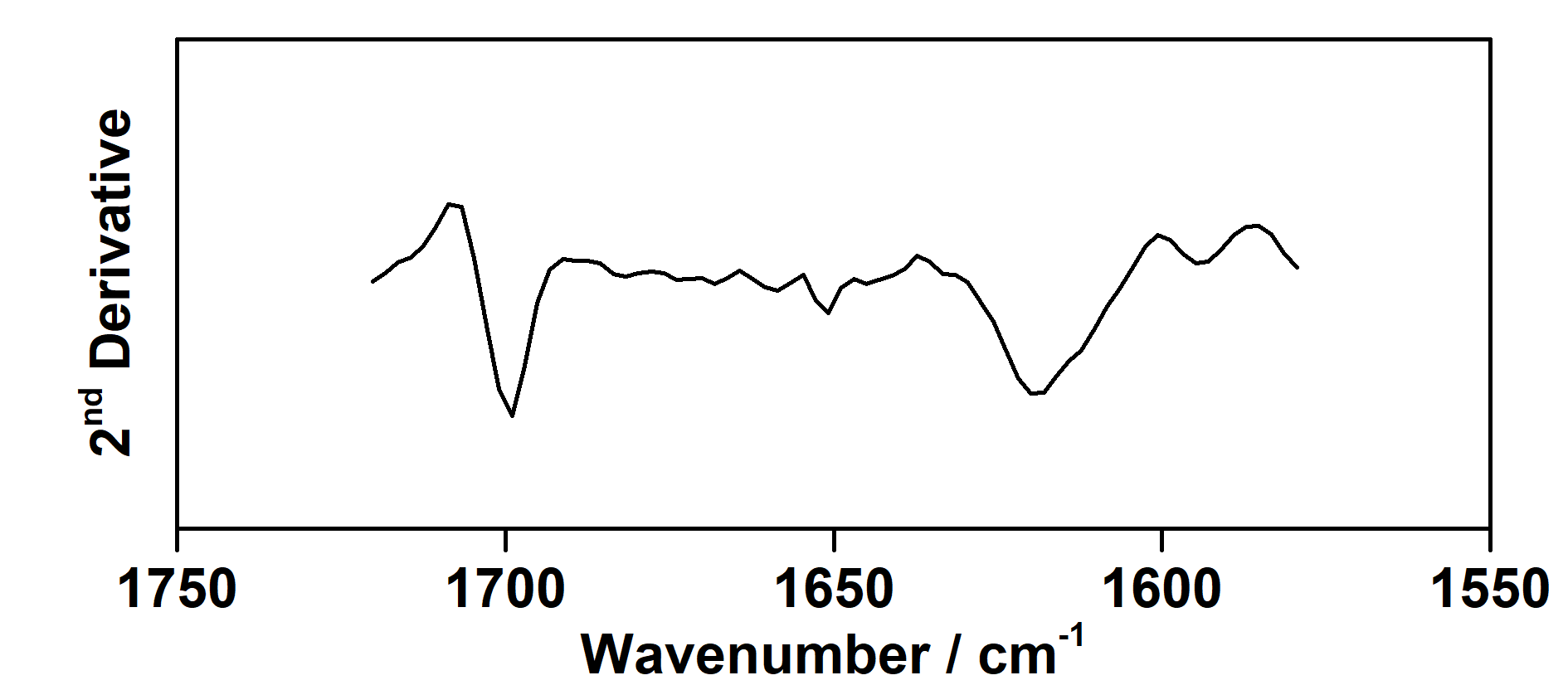

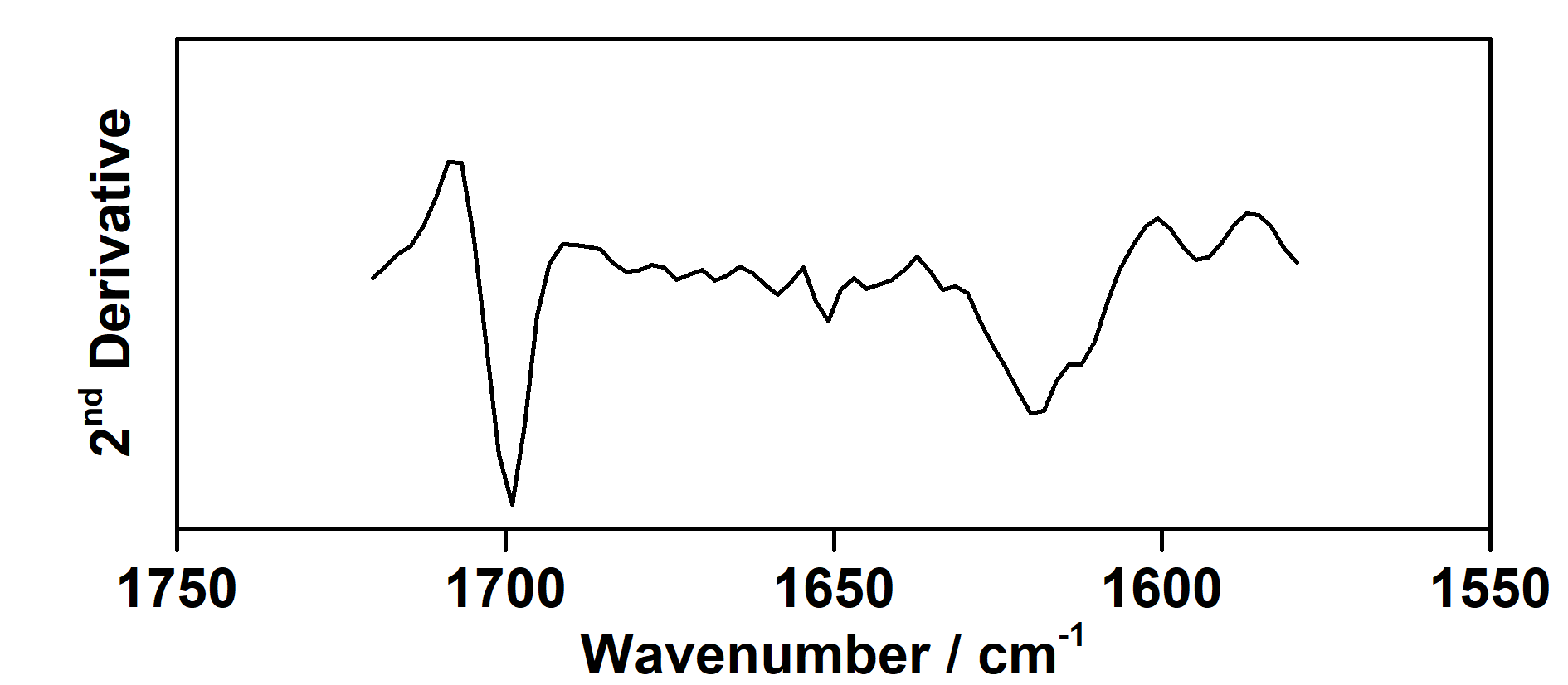


**Figure S5** Representative FTIR and second derivative spectra of aligned NSB fibres in the range of 1720-1580 cm^-1^. Deconvolution of amide I band of (A) NSB-7.5x, (B) NSB-10.0x, (C) NSB-12.5x and (D) NSB-15.0x fibres, showing changes in ratios of secondary structures in increasingly post-drawn fibres.


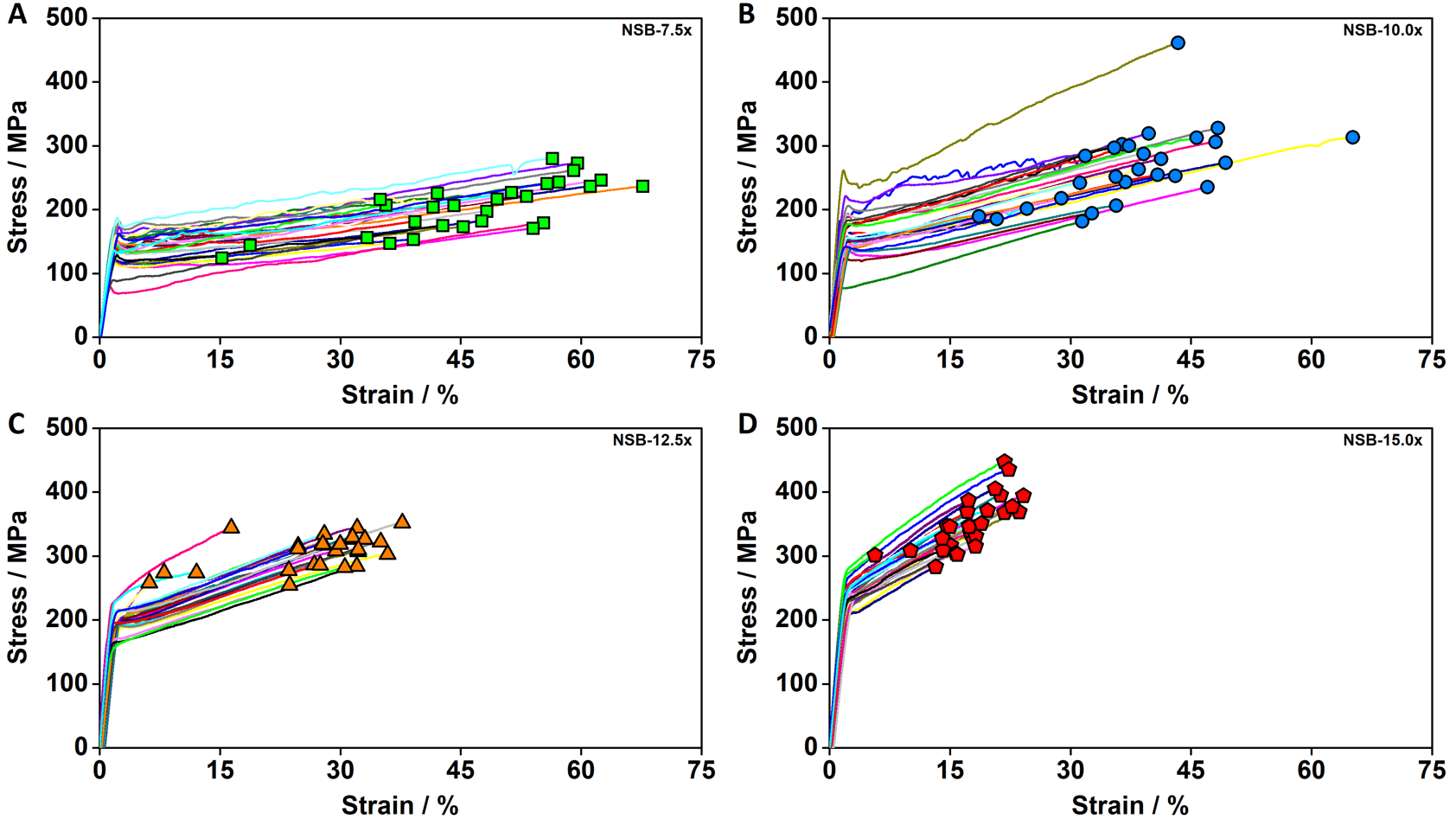


**Figure** **S6** Quasi-static tensile stress–strain curves of NSB fibres fabricated at various draw-ratios: (A) NSB-7.5x, (B) NSB-10.0x, (C) NSB-12.5x and (D) NSB-15.0x. Briefly, the utilisation of enhanced draw-ratios resulted in increased fibre stiffness properties, along with increased consistency in fracture strength and extension properties, and vice versa.

**Table** **S2** Properties and references of artificially spun single & solid silk fibres reported in literature.^7,8^

| **Process** | **Protein concentration** | | **Fibre Properties** | | | | | **Reference** |
| --- | --- | --- | --- | --- | --- | --- | --- | --- |
|  | **% w/w** | **% w/v** | **Diameter** | **Stiffness** | **Strength** | **Extensibility** | **Toughness** |  |
|  |  |  | **µm** | **GPa** | **MPa** | **%** | **MJ/m^3^** |  |
| RSF Wet Spinning | 20.0 | / | 118.5 | 6.7 | 130.0 | 11.0 | 12.9 | Matsumoto *et al.*^9^ |
|  | 10.0 | / | 40.0 | 5.3 | 321.2 | 16.1 | 37.6 | Yao *et al.*^10^ |
|  | 10.0 | / | 40.0 | 5.2 | 193.0 | 19.0 | 28.2 | Zhao *et al.*^11^ |
|  | 15.6 | / | 189.0 | 4.1 | 103.8 | 40.0 | 38.0 | Um *et al.*^12^ |
|  | 15.6 | / | 119.0 | 5.5 | 257.5 | 16.4 | 30.6 | Um *et al.*^12^ |
|  | 13.0 | / | 18.5 | 7.2 | 120.0 | 35.0 | 38.9 | Marsano *et al.*^13^ |
|  | 13.0 | / | 35.0 | 39.9 | 1077.3 | 29.3 | 257.8 | Ha *et al.*^14^ |
|  | 13.0 | / | 21.0 | 43.2 | 959.0 | 18.1 | 156.7 | Ha *et al.*^14^ |
|  | 15.6 | / | 220.0 | 4.9 | 269.4 | 19.5 | 38.7 | Lee *et al.*^15^ |
|  | 17.0 | / | 73.0 | 5.3 | 127.0 | 12.7 | 20.3 | Corsini *et al.*^16^ |
|  | 12.3 | / | 100.0 | 7.2 | 285.1 | 14.0 | 30.4 | Ki *et al.*^17^ |
|  | 12.0 | / | 40.0 | 4.3 | 400.5 | 20.7 | 51.3 | Zhu *et al.*^18^ |
|  | 29.0 | / | 20.0 | 6.0 | 128.8 | 7.6 | 6.8 | Sohn *et al.*^19^ |
|  | 17.0 | / | 41.0 | 13.4 | 313.6 | 8.5 | 20.5 | Plaza *et al.*^20^ |
|  | 17.0 | / | 47.0 | 5.1 | 172.4 | 48.4 | 55.5 | Plaza *et al.*^20^ |
|  | 15.0 | / | 10.8 | 12.5 | 450.0 | 27.7 | 100.6 | Zhou *et al.*^21^ |
|  | 17.0 | / | 18.4 | 7.4 | 257.6 | 35.3 | 51.9 | Plaza *et al.*^22^ |
|  | 20.0 | / | 100.0 | 11.2 | 221.0 | 30.0 | 46.4 | Ling *et al.*^23^ |
|  | 12.0 | / | 12.8 | 6.9 | 470.4 | 38.6 | 105.3 | Zhang *et al.*^24^ |
|  | 15.0 | / | 15.0 | 18.9 | 450.0 | 27.3 | 91.0 | Fang *et al.*^25^ |
|  | 13.0 | / | 25.0 | 37.8 | 98.0 | 58.9 | 53.5 | Chen *et al.*^26^ |
|  | 16.0 | / | 9.0 | 11.0 | 330.0 | 22.0 | 50.0 | Madurga *et al.*^27^ |
| RSF Dry Spinning | 20.0 | / | 5.7 | 6.2 | 301.5 | 35.8 | 104.8 | Wei *et al.*^28^ |
|  | 20.0 | / | 6.4 | 5.8 | 295.2 | 74.8 | 155.9 | Wei *et al.*^28^ |
|  | 50.0 | / | 10.0 | 11.1 | 337.7 | 24.6 | 55.8 | Sun *et al.*^29^ |
|  | 40.0-60.0 | / | 6.3 | 8.8 | 357.3 | 34.1 | 86.5 | Jin *et al.*^30^ |
|  | 50.0 | / | 17.0 | 19.0 | 614.0 | 27.0 | 136.4 | Luo *et al.*^31^ |
|  | 20.0-25.0 | / | 20.0 | 8.8 | 333.0 | 35.1 | 90.9 | Yue *et al.*^32^ |
|  | 38.0-47.0 | / | 9.0 | 9.4 | 541.3 | 19.3 | 76.4 | Peng *et al.*^33^ |
|  | 5.0 | / | 50.5 | 11.0 | 93.0 | 4.5 | 2.4 | Ling *et al.*^34^ |
| Recombinant Wet Spinning | 23.0 | / | 20.0 | 13.2 | 269.6 | 43.3 | 101.4 | Lazaris et al.^35^ |
|  | 25.0-30.0 | / | 15.8 | 1.1 | 49.6 | 15.8 | 10.6 | Teulé *et al.*^36^ |
|  | 10.0-12.0 | / | 74.1 | 0.4 | 49.5 | 3.6 | 4.7 | Brooks *et al.*^37^ |
|  | / | N/A | 46.0 | 4.5 | 246.7 | 50.6 | 91.7 | Elices *et al.*^38^ |
|  | / | 30.0 | 17.4 | 5.7 | 132.5 | 22.8 | 23.7 | An *et al.*^39^ |
|  | / | 26.0-27.0 | 28.3 | 4.4 | 127.5 | 52.3 | 54.6 | Teulé *et al.*^40^ |
|  | / | 26.0-27.0 | 14.0 | 3.8 | 96.2 | 29.6 | 22.6 | Teulé *et al.*^40^ |
|  | / | 30.0 | 29.1 | 3.4 | 37.6 | 53.9 | 17.4 | An *et al.*^41^ |
|  | / | 30.0 | 29.1 | 4.3 | 59.6 | 4.8 | 2.5 | An *et al.*^41^ |
|  | / | 20.0 | 24.5 | 3.9 | 121.9 | 18.0 | 17.4 | Gnesa *et al.*^42^ |
|  | / | 20.0 | 30.5 | 2.6 | 95.1 | 25.0 | 20.7 | Gnesa *et al.*^42^ |
|  | / | 15.0 | 15.1 | 4.0 | 150.6 | 84.5 | 89.1 | Adrianos *et al.*^43^ |
|  | / | 8.0-10.0 | 10.0 | 9.3 | 308.0 | 9.6 | 24.4 | Lin *et al.*^44^ |
|  | / | 45.0-60.0 | 31.5 | 2.9 | 53.5 | 18.0 | 9.3 | Albertson *et al.*^45^ |
|  | / | 45.0-60.0 | 36.0 | 1.6 | 39.0 | 181.3 | 59.3 | Albertson *et al.*^45^ |
|  | / | 10.0-17.0 | 27.0 | 4.0 | 370.0 | 110.0 | 189.0 | Heidebrecht *et al.*^46^ |
|  | / | 12.0 | 34.0 | 4.0 | 62.3 | 3.5 | 1.6 | Peng *et al.*^47^ |
|  | / | 10.0-17.0 | 14.0 | 8.4 | 286.2 | 18.3 | 37.7 | Peng *et al.*^47^ |
|  | / | 50.0 | 12.0 | 6.0 | 162.0 | 37.0 | 45.0 | Andersson *et al.*^48^ |
|  | / | 17.0 | 5.7 | 13.7 | 1031.0 | 18.0 | 114.0 | Bowen *et al.*^8^ |

*Note:* Only studies with complete & comparable data sets (diameter and/or cross-section area, engineering tensile stress, engineering tensile strain, toughness) of artificially spun single & solid silk fibres are listed.


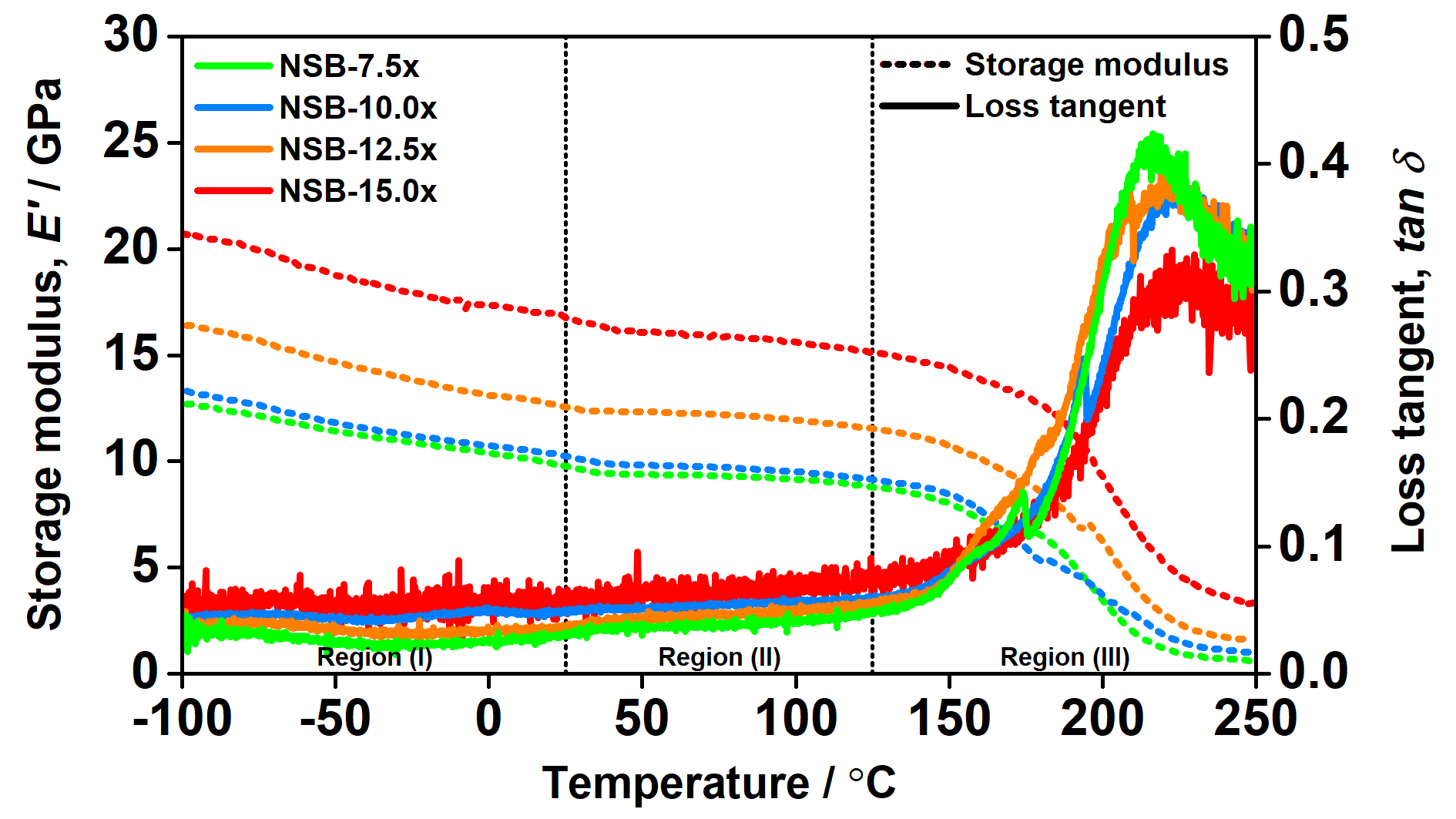


**Figure** **S7** Representative DMTA profiles of NSB fibres, showing the changes of the storage modulus and loss tangent as a function of temperature. Briefly, the full temperature range of the DMTA profiles can be divided into three regions: (I) The storage modulus of all NSB fibres decreased with increasing temperature from -100 to 25°C, where the NSB-7.5x, NSB-10.0x, NSB-12.5x and NSB-15.0x fibres featured a reference storage modulus of 9.8 GPa, 10.2 GPa, 12.6 GPa and 16.8 GPa at 25°C, respectively. With respect to the loss tangent curves, all NSB fibres demonstrated only minor transition effects in the low-temperature region. (II) The storage modulus of all NSB fibres decreased minimally with increasing temperature from 25 to 125°C. Regarding to the loss tangent curves, all NSB fibres presented only minor gradual changes. (III) The storage modulus of all NSB fibres decreased sharply from 125 to 250°C, where the NSB-7.5x, NSB-10.0x, NSB-12.5x and NSB-15.0x fibres featured a glass transition temperature at the loss tangent peak of 216°C, 219.5°C, 225.5°C and 228.5°C, respectively. Correspondently, the NSB-7.5x, NSB-10.0x, NSB-12.5x and NSB-15.0x fibres featured loss tangent peaks of 0.42, 0.39, 0.37 and 0.32, respectively. With this respect, smaller loss tangent peaks suggests a reduced amount of amorphous/disordered structures in the silk structure, which also explains the increase in glass transition.^49,50^ In addition, NSB-7.5x, NSB-10.0x and NSB-12.5x fibres presented discrete loss tangent shoulders in the temperature range of 150 to 200°C, which can be associated with more disordered silk structures, while NSB-15.0x fibres showed no loss tangent peaks in high-temperature regions. In comparison, standard native silk fibres from *Bombyx Mori* feature a relative similar storage modulus trend over the full temperature range and were characterised with a reference storage modulus of 9.2 GPa at 25°C, as previous work illustrated.^49,50^ However, native silk fibres were characterised with distinct loss tangent peaks in the temperature region (I) at -60°C and region (II) at 60°C, which can be assigned to the *β*-relaxation of silk fibroin and water-protein interactions, respectively.^49,50^


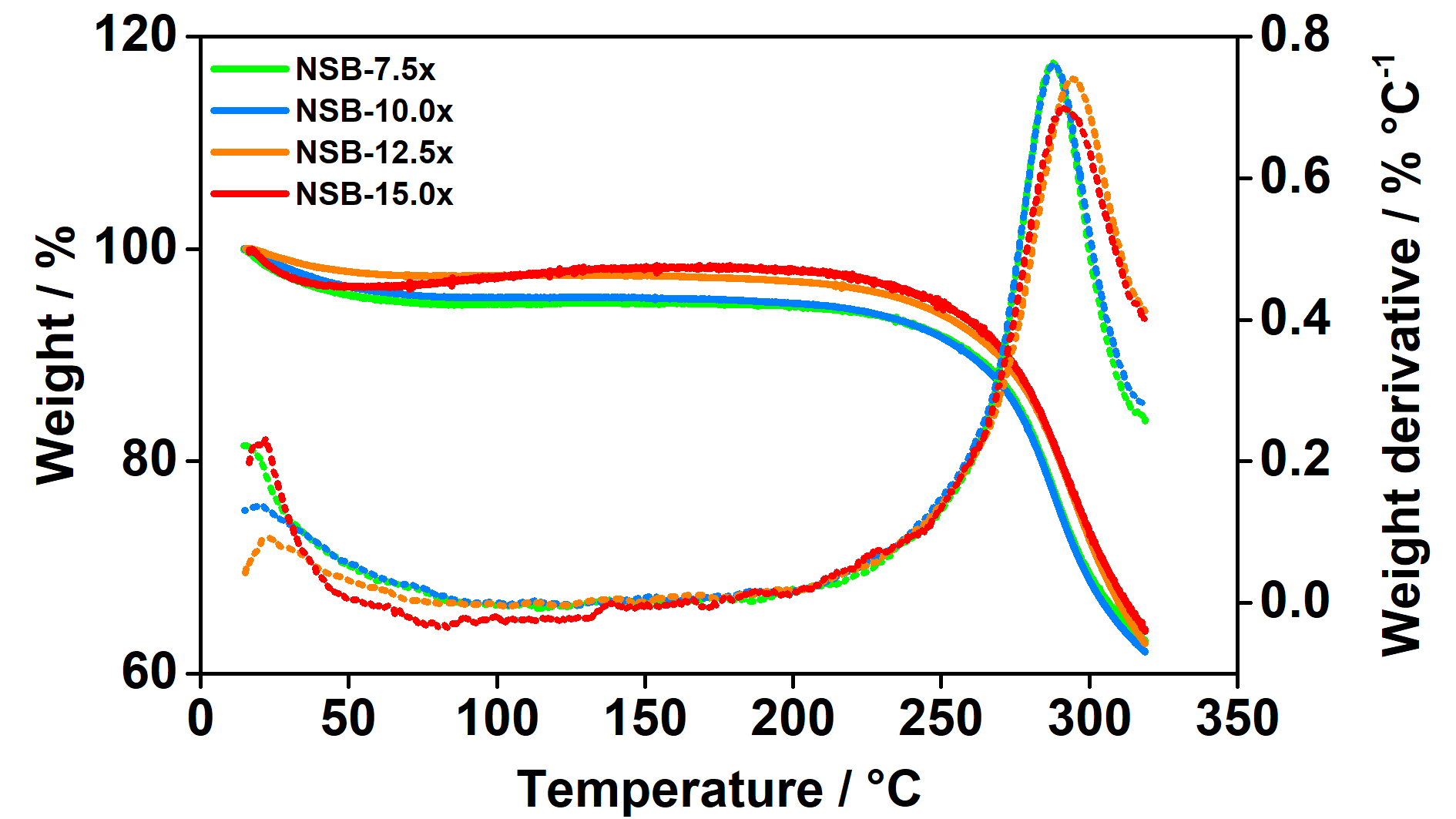


**Figure** **S8** Representative TGA curves of NSB fibres. Briefly, the water content in NSB fibres was estimated by measuring the weight loss up to 120 °C, in which the NSB-7.5x, NSB-10.0x, NSB-12.5x and NSB-15.0x fibres were characterised with a water content of 5.07 ± 0.51%, 4.52 ± 0.14%, 4.21 ± 1.31% and 2.33 ± 0.38%, respectively. In comparison, native silk and degummed silk fibres from *Bombyx mori* featured a relative similar water content of ~6% and ~4.5%, respectively.^50^ The TGA results imply a strong link between draw-ratio and water content, which correlates with previous research results.^49,50^ A greater water content in lower draw-ratio NSB fibres can be associated with an increased presence of disordered silk and amorphous structures, and correspondingly has an effect on the thermal decomposition characteristics of these fibres.^49,50^ In this respect, the NSB-7.5x, NSB-10.0x, NSB-12.5x and NSB-15.0x fibres obtained a weight loss of 31.73 ± 1.18%, 31.32 ± 0.58%, 29.31 ± 1.87% and 27.89 ± 1.14% at 300°C, respectively.

**References**

1. Boulet-Audet, M., Vollrath, F. & Holland, C. Identification and classification of silks using infrared spectroscopy. *J. Exp. Biol.* **218,** 3138–3149 (2015).

2. Laity, P. R. & Holland, C. Native Silk Feedstock as a Model Biopolymer: A Rheological Perspective. *Biomacromolecules* **17,** 2662-2671 (2016).

3. Laity, P. R., Gilks, S. E. & Holland, C. Rheological behaviour of native silk feedstocks. *Polymer* **67,** 28–39 (2015).

4. Holland, C., Terry, A. E., Porter, D. & Vollrath, F. Natural and unnatural silks. *Polymer* **48,** 3388–3392 (2007).

5. Holland, C., Terry, A. E., Porter, D. & Vollrath, F. Comparing the rheology of native spider and silkworm spinning dope. *Nat. Mater.* **5,** 870–874 (2006).

6. Laity, P. R. & Holland, C. The Rheology behind Stress-Induced Solidification in Native Silk Feedstocks. *Int. J. Mol. Sci.* **17,** 1812 (2016).

7. Koeppel, A. & Holland, C. Progress and Trends in Artificial Silk Spinning: A Systematic Review. *ACS Biomaterials Science and Engineering* **3,** 226–237 (2017).

8. Bowen, C. H. *et al.* Recombinant Spidroins Fully Replicate Primary Mechanical Properties of Natural Spider Silk. *Biomacromolecules* **19,** 3853–3860 (2018).

9. Matsumoto, K., Uejima, H., Iwasaki, T., Sano, Y. & Sumino, H. Studies on regenerated protein fibers .3. Production of regenerated silk fibroin fiber by the self-dialyzing wet spinning method. *J. Appl. Polym. Sci.* **60,** 503–511 (1996).

10. Yao, J., Masuda, H., Zhao, C. & Asakura, T. Artificial spinning and characterization of silk fiber from bombyx mori silk fibroin in hexafluoroacetone hydrate. *Macromolecules* **35,** 6–9 (2002).

11. Zhao, C. C., Yao, J., Masuda, H., Kishore, R. & Asakura, T. Structural characterization and artificial fiber formation of bombyx mori silk fibroin in hexafluoro-Iso-Propanol solvent system. *Biopolymers* **69,** 253–259 (2003).

12. Um, I. C. *et al.* Wet spinning of silk polymer: I. Effect of coagulation conditions on the morphological feature of filament. *Int. J. Biol. Macromol.* **34,** 89–105 (2004).

13. Marsano, E. *et al.* Wet spinning of Bombyx mori silk fibroin dissolved in N-methyl morpholine N-oxide and properties of regenerated fibres. *Int. J. Biol. Macromol.* **37,** 179–188 (2005).

14. Ha, S.-W., Tonelli, A. E. & Hudson, S. M. Structural studies of Bombyx mori silk fibroin during regeneration from solutions and wet fiber spinning. *Biomacromolecules* **6,** 1722–1731 (2005).

15. Lee, K. H., Baek, D. H., Ki, C. S. & Park, Y. H. Preparation and characterization of wet spun silk fibroin/poly(vinyl alcohol) blend filaments. *Int. J. Biol. Macromol.* **41,** 168–172 (2007).

16. Corsini, P. *et al.* Influence of the draw ratio on the tensile and fracture behavior of NMMO regenerated silk fibers. *J. Polym. Sci. Part B Polym. Phys.* **45,** 2568–2579 (2007).

17. Ki, C. S. *et al.* Dissolution and wet spinning of silk fibroin using phosphoric acid/formic acid mixture solvent system. *J. Appl. Polym. Sci.* **105,** 1605–1610 (2007).

18. Zhu, Z., Imada, T. & Asakura, T. Preparation and characterization of regenerated fiber from the aqueous solution of Bombyx mori cocoon silk fibroin. *Mater. Chem. Phys.* **117,** 430–433 (2009).

19. Sohn, S. & Gido, S. P. Wet-Spinning of Osmotically Stressed Silk Fibroin. *Biomacromolecules* **10,** 2086–2091 (2009).

20. Plaza, G. R. *et al.* Old Silks Endowed with New Properties. *Macromolecules* **42,** 8977–8982 (2009).

21. Zhou, G., Shao, Z., Knight, D. P., Yan, J. & Chen, X. Silk Fibers Extruded Artificially from Aqueous Solutions of Regenerated *Bombyx mori* Silk Fibroin are Tougher than their Natural Counterparts. *Adv. Mater.* **21,** 366–370 (2009).

22. Plaza, G. R. *et al.* Correlation between processing conditions, microstructure and mechanical behavior in regenerated silkworm silk fibers. *J. Polym. Sci. Part B Polym. Phys.* **50,** 455–465 (2012).

23. Ling, S., Zhou, L., Zhou, W., Shao, Z. & Chen, X. Conformation transition kinetics and spinnability of regenerated silk fibroin with glycol, glycerol and polyethylene glycol. *Mater. Lett.* **81,** 13–15 (2012).

24. Zhang, F. *et al.* Regeneration of high-quality silk fibroin fiber by wet spinning from CaCl2–formic acid solvent. *Acta Biomater.* **12,** 139–145 (2015).

25. Fang, G. *et al.* Insights into Silk Formation Process: Correlation of Mechanical Properties and Structural Evolution during Artificial Spinning of Silk Fibers. *ACS Biomater. Sci. Eng.* **2,** 1992–2000 (2016).

26. Chen, Z. *et al.* Programing Performance of Silk Fibroin Materials by Controlled Nucleation. *Adv. Funct. Mater.* **26,** 8978–8990 (2016).

27. Madurga, R. *et al.* Production of High Performance Bioinspired Silk Fibers by Straining Flow Spinning. *Biomacromolecules* **18**, 1127–1133 (2017).

28. Wei, W., Zhang, Y., Shao, H. & Hu, X. Posttreatment of the dry-spun fibers obtained from regenerated silk fibroin aqueous solution in ethanol aqueous solution. *J. Mater. Res.* **26,** 1100–1106 (2011).

29. Sun, M., Zhang, Y., Zhao, Y., Shao, H. & Hu, X. The structure–property relationships of artificial silk fabricated by dry-spinning process. *J. Mater. Chem.* **22,** 18372 (2012).

30. Jin, Y., Zhang, Y., Hang, Y., Shao, H. & Hu, X. A simple process for dry spinning of regenerated silk fibroin aqueous solution. *J. Mater. Res.* **28,** 2897–2902 (2013).

31. Luo, J. *et al.* Tough silk fibers prepared in air using a biomimetic microfluidic chip. *Int. J. Biol. Macromol.* **66,** 319–324 (2014).

32. Yue, X. *et al.* A novel route to prepare dry-spun silk fibers from CaCl2–formic acid solution. *Mater. Lett.* **128,** 175–178 (2014).

33. Peng, Q., Shao, H., Hu, X. & Zhang, Y. Role of humidity on the structures and properties of regenerated silk fibers. *Prog. Nat. Sci. Mater. Int.* **25,** 430–436 (2015).

34. Ling, S. *et al.* Polymorphic regenerated silk fibers assembled through bioinspired spinning. *Nat. Commun.* **8,** 1387 (2017).

35. Lazaris, A. *et al.* Spider Silk Fibers Spun from Soluble Recombinant Silk Produced in Mammalian Cells. *Science* **295,** 472 (2002).

36. Teulé, F., Furin, W. A., Cooper, A. R., Duncan, J. R. & Lewis, R. V. Modifications of spider silk sequences in an attempt to control the mechanical properties of the synthetic fibers. *J. Mater. Sci.* **42,** 8974–8985 (2007).

37. Brooks, A. E. *et al.* Properties of Synthetic Spider Silk Fibers Based on Argiope aurantia MaSp2. *Biomacromolecules* **9,** 1506–1510 (2008).

38. Elices, M. *et al.* Bioinspired Fibers Follow the Track of Natural Spider Silk. *Macromolecules* **44,** 1166–1176 (2011).

39. An, B., Hinman, M. B., Holland, G. P., Yarger, J. L. & Lewis, R. V. Inducing β-Sheets Formation in Synthetic Spider Silk Fibers by Aqueous Post-Spin Stretching. *Biomacromolecules* **12,** 2375–2381 (2011).

40. Teulé, F. *et al.* Combining flagelliform and dragline spider silk motifs to produce tunable synthetic biopolymer fibers. *Biopolymers* **97,** 418–31 (2012).

41. An, B. *et al.* Reproducing Natural Spider Silks’ Copolymer Behavior in Synthetic Silk Mimics. *Biomacromolecules* **13,** 3938–3948 (2012).

42. Gnesa, E. *et al.* Conserved C-Terminal Domain of Spider Tubuliform Spidroin 1 Contributes to Extensibility in Synthetic Fibers. *Biomacromolecules* **13,** 304–312 (2012).

43. Adrianos, S. L. *et al.* Nephila clavipes Flagelliform Silk-Like GGX Motifs Contribute to Extensibility and Spacer Motifs Contribute to Strength in Synthetic Spider Silk Fibers. *Biomacromolecules* **14,** 1751–1760 (2013).

44. Lin, Z., Deng, Q., Liu, X.-Y. & Yang, D. Engineered Large Spider Eggcase Silk Protein for Strong Artificial Fibers. *Adv. Mater.* **25,** 1216–1220 (2013).

45. Albertson, A. E., Teulé, F., Weber, W., Yarger, J. L. & Lewis, R. V. Effects of different post-spin stretching conditions on the mechanical properties of synthetic spider silk fibers. *J. Mech. Behav. Biomed. Mater.* **29,** 225–234 (2014).

46. Heidebrecht, A. *et al.* Biomimetic Fibers Made of Recombinant Spidroins with the Same Toughness as Natural Spider Silk. *Adv. Mater.* **27,** 2189–2194 (2015).

47. Peng, Q. *et al.* Recombinant spider silk from aqueous solutions via a bio-inspired microfluidic chip. *Sci. Rep.* **6,** 36473 (2016).

48. Andersson, M. *et al.* Biomimetic spinning of artificial spider silk from a chimeric minispidroin. *Nat. Chem. Biol.* **13,** 262–264 (2017).

49. Guan, J., Porter, D. & Vollrath, F. Thermally Induced Changes in Dynamic Mechanical Properties of Native Silks. *Biomacromolecules* **14,** 930-937 (2013).

50. Mortimer, B., Guan, J., Holland, C., Porter, D. & Vollrath, F. Linking naturally and unnaturally spun silks through the forced reeling of Bombyx mori. *Acta Biomater.* **11,** 247–255 (2015).
